# A Systematic Literature Review on Biometric Authentication in Mobile Banking

**DOI:** 10.12688/f1000research.173855.1

**Published:** 2026-01-06

**Authors:** Hasan Naji Ali, SUFYAN SALIM MAHMOOD AL-Dabbagh

**Affiliations:** 1 Computer Science, University of Mosul College of Computer Sciences and Mathematics, Mosul, Nineveh Governorate, Iraq; 2 Computer Science, Tikrit University College of Computer Science and Mathematics, Tikrit, Saladin Governorate, Iraq; 3 Cybersecurity, University of Mosul College of Computer Sciences and Mathematics, Mosul, Nineveh Governorate, Iraq

**Keywords:** mobile banking, biometric authentication, user authentication, usability and privacy, multi-factor authentication, cybersecurity threats

## Abstract

As mobile banking continues to grow at an exponential rate, the financial industry is faced with a critical challenge: How to keep user credentials secure without compromising on efficiency. Password-based authentication is still dominant but has major limitations which compromise both security and user experience. These systems are susceptible to the most common attack vectors such as phishing, malware and man-in-the-middle attacks, especially if users are using weak passwords or sharing passwords. Additionally, mobile devices have limited input interfaces that are frequently sources of frustration and error. As a result, there is increasing interest in other more secure and convenient alternatives such as biometric and multi-factor authentication (MFA) to mitigate the inherent weaknesses of password-based systems. This systematic literature review, which covers studies from 2020 to 2025, provides a critical review of biometric authentication methods used in mobile banking. It analyses existing approaches, security risks and implementation practices adopted by major banks across the world. While biometric systems are more secure and user friendly than traditional systems, they also introduce new challenges in terms of privacy, spoofing and regulatory compliance. The review gives a detailed overview of the current advances, key issues, and emerging research directions, which will give valuable insight to the development of secure and easy-to-use authentication systems in mobile banking.

## 1. Introduction

**Figure 1.**
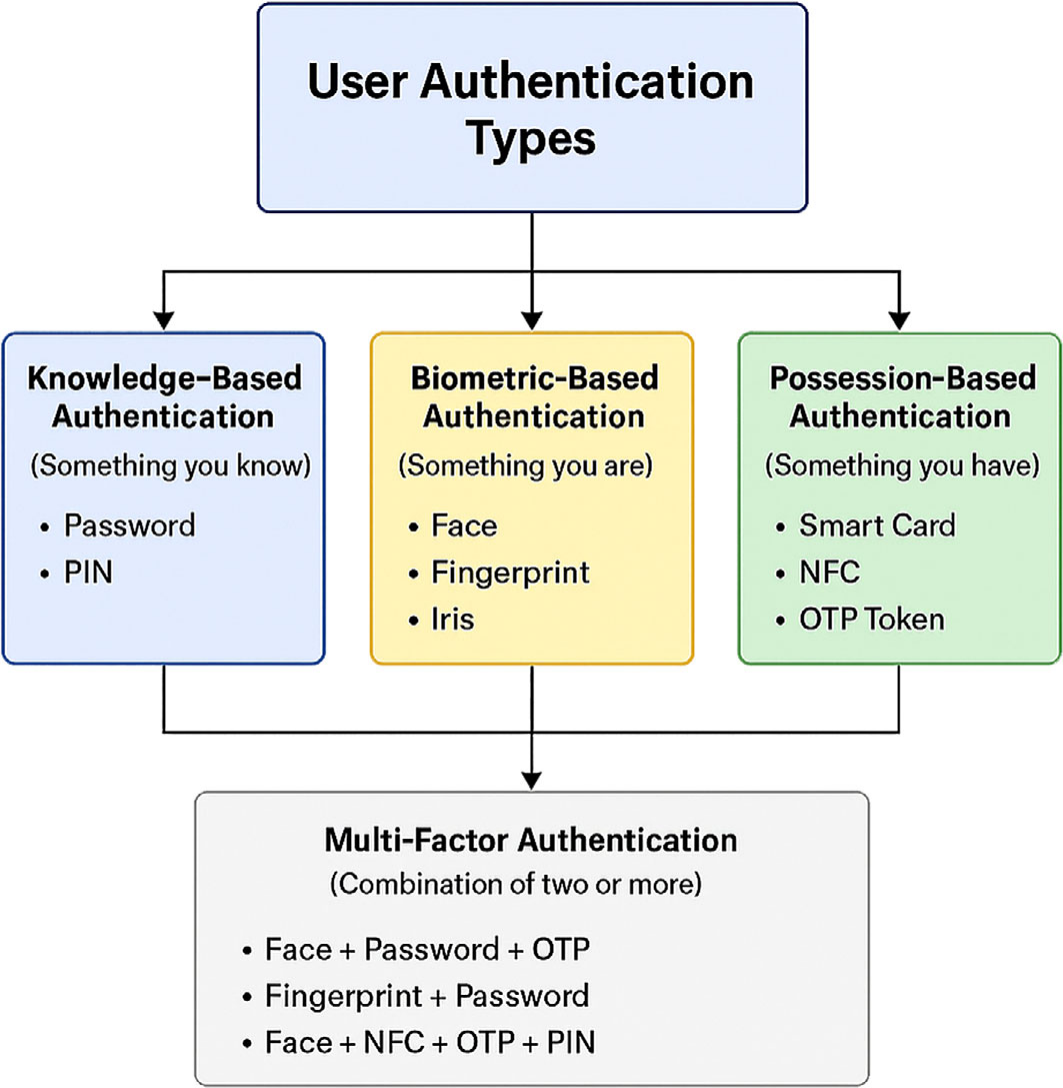
Classification of general user authentication methods categorized as knowledge-based, possession-based, and biometric-based factors.

Modern financial systems rely heavily on mobile banking because smartphones have become the standard, and digital dependence has grown worldwide. Mobile banking has other names, such as e-banking, online payments, online banking and internet banking. The electronic payments system provides bank clients and financial institution users with the ability to conduct transactions through the internet. Digital transformation through mobile technology simultaneously produces substantial security threats because cyber attackers now focus on stealing sensitive financial data and monitoring financial activities. A combination of passwords with personal identification numbers (PINs) and security questions proves inadequate as authentication methods because they remain susceptible to various attacks, such as brute force attacks alongside phishing and social engineering attacks and credential stuffing. ^1, 2^ The growing need for safer and easier-to-use authentication systems results directly from these system vulnerabilities. Biometric authentication has arisen as a disruptive solution that uses individual physical or behavioral characteristics such as fingerprints, facial recognition, voice, irises, signatures, etc. to authenticate users with enhanced safety and precision. Online banking entry methods through which users prove their identity for platform access are known as user authentication. The authentication approaches of mobile banking systems protect both financial service access and users from fraudulent activities. where user authentication verifies the actual identity of a declared person or application or procedure handling user requests. System access control relies on authentication technology, which verifies that user credentials exist in authorized user databases or data authentication servers. Organizations protect their networks through authentication because this procedure allows only verified users (or automated processes) to reach secure assets, including systems, networks, databases, websites and remote applications. There are three principal authentication system types ^3^ as illustrated in Figure 1.

1. **Something you know:** Refer to knowledge-based authentication (KBA) User authentication methods depend exclusively on information that only users possess. At present, passwords together with PINs function as standard authentication methods, although they face dangers from phishing schemes, brute-force attacks and password exposure incidents. ^4^ User security questions function as a backup authentication process yet become vulnerable when attackers acquire access. ^5^
2. **Something you have**: Also refer to possession-based authentication (PBA), users must possess hardware or equipment to use these authentication methods. One time password (OTP) ^6, 7^: Sent via short message send (SMS) messages, emails, or authenticator apps. Devices used for extra security face potential vulnerabilities when attackers conduct SIM swap attacks against them. ^8^ Users can use hardware tokens together with smart cards to achieve maximum security; however, they require the physical possession of additional hardware devices.
3. **Something you are**: Also referring to biometric-based authentication (BBA), is a security method based on an individual’s unique biometric and/or biometric trait that can be used to verify the identity of the individual. For example, fingerprints, facials, iris patterns, and hand and voice recognition are common biometric identifiers. These traits are distinctive and extremely difficult to reproduce; thus, the BBA is much more secure than typical authentication methods such as passwords or PINs. ^9^ The convenience of BBA is that the users do not need to remember a password much less often to bring physical tokens. ^10– 12^ The AI-driven verification system identifies people by their faces, although it delivers insufficient results under poor lighting conditions and when users wear masks. ^13^ Another challenge, however, regarding the implementation of the BBA is that of privacy as well as protection of biometric data. Since biometric traits that are compromised cannot be switched for new passwords, long-term security becomes an issue. However, despite these concerns, such as the ability of adversaries to learn the distribution of nonbits, BBA technology has been widely adopted in many fields, including mobile devices, financial services and national security, because of its strong ability to enhance the process of authentication. ^14^
4. **Multi-factor authentication (MFA):** This method combines two or more authentication methods for enhanced security. Users must authenticate through KBA password entry on a mobile banking application and receive receipt of an OTP. ^15, 16^ Security increases when the system maintains multiple authentication methods because compromised individual factors do not authorize unauthorized access. Additional extra security layers are appended when using biometric authentication, such as fingerprints, facial recognition, irises, voice, hands/palms, ^17^ etc. Continuous authentication delivers two forms of protection by evaluating keyboard typing patterns and device motion along with swipe gestures. AI-based risk assessment conducts continuous assessments of user engagements to discover doubtful activities in real time. ^18, 19^ Table 1 present analysis of user authentication approaches includes descriptions, strengths, weaknesses, and typical use cases.

**Table 1.** Comparative analysis of major types of authentications based on their description, advantages, disadvantages and major applications.

Authentication method	Description	Strengths	Weaknesses	Common use cases	Ref.
**KBA**	Users establish personal secret characters.	Simple, cost-effective, widely supported.	Vulnerable to brute force, phishing, and weak passwords.	Logins for websites, apps, and systems.	^20, 21^
**PBA**	Uses physical or digital tokens (e.g., OTPs, smart cards).	Adds an extra layer of security, time-sensitive codes.	Tokens can be lost or stolen, requires users to carry a device.	Online banking, corporate networks, VPNs.	^22^
**BBA**	Uses unique physical traits (e.g., fingerprints, facial recognition).	Difficult to replicate, convenient, and secure.	Privacy concerns, potential for false positives/negatives, high implementation cost.	Smartphones, high-security facilities, banking.	^23, 24^
**MFA**	Combines two or more authentication factors (e.g., PIN + OTP or biometric).	Highly secure, reduces risk of unauthorized access.	It can be inconvenient if factors are not readily available.	Banking, email, and enterprise systems.	^25^

### 1.1 Motivation

The rapid increase in mobile banking has exposed financial facilities to well advanced cyber risks, and at the same time, it made the processes rather convenient. Password-based techniques of authentication are exposed to several attacks such as phishing attacks, brute-force attacks, and face issues in areas of memorability and limits of mobile operating environment. Therefore, an incentive to approach biometric solutions that will provide higher security levels and user experience is present in the sphere of mobile banking.

By searching previous studies for the period (2020-2025), we did not find a systematic literature review that focused specifically on the uses of BBA in mobile banking. There are some studies that focused on multi-factor authentication, while others focus on the use of all authentication methods in general and their uses in online banking. Therefore, in this research, we focused on studies that examined the use of biometric authentication in mobile banking.

So, the main contributions of this study are summarized as follows:

• Comprehensive systematic literature review (SLR): This study provides a detailed of systematic literature review based-on the PRISMA methodology and summarizes recent studies (2020–2025) on biometric authentication, especially focusing on mobile banking systems.
• Design a taxonomy that includes all the biometric authentication methods used in previous studies, making it easier for the researcher to gain knowledge about these methods that are used in mobile banking.
• Analysis of Security Threats: Among the security threats that are identified and discussed in the context of both biometric authentication methods, the study lists such critical ones as biometric spoofing, malware, phishing, social engineering, a man-in-the-middle attack, etc. Besides, feasible measures against mitigation are also offered to make the institutions have an insight into how to persevere with the given threats.
• Survey of biometric authentication methods that are used in global banking practices: This study evaluates the authentication systems used by the major banks across the world, which refers to state-of-the-art biometric solutions in the real-life financial situation, filling the gap between academic evidence and industrial practice.
• Insight into usability and user perception: This paper discusses some of the usability and user perception issues in the application of biometrics to mobile banking, such as sensor reliability, privacy of stored biometric data, device and hardware constraints, and the security vs. convenience trade-off. It provides guidance for financial institutions and policy makers in creating authentication systems that are secure as well as convenient to use.
• Future Research Directions: Lastly, this study identifies key gaps in current practices and recommends future work on adaptive and context-aware authentication, privacy-preserving biometric security, emerging threat models, and AI-driven protection. It serves as a valuable guide for advancing secure and user-friendly mobile banking authentication in both academia and industry.

### 1.2 Intended audience

The present manuscript is aimed at a wide audience of both academic researchers and industry practitioners. To the academic community, the research can help in offering a synthesis of the state of the art biometric authentication methods in mobile banking to serve as a reference point when carrying out further studies in the research subject. The review highlights to financial institutions, banking professionals, and policymakers some practical challenges, new security threats, and usability challenges that must be addressed to ensure that mobile banking can become more secure. The paper can also be of practical use to developers and system designers interested in deploying serviceable and easy-to-use authentication systems in financial applications.

The rest of this paper proceeds as follows: **Section 2** presents an overview of mobile banking, biometric authentication, role of biometrics in mobile banking and overall rating, **Section 3** presents the research methodology, detailing criteria and process we applied in choosing and assessing the academic papers that are collected. **Section 4** provides a comprehensive analysis of the academic papers that are gathered, discussing methods used in mobile banking, threat facing mobile banking and biometric authentication methods in leading banks and challenges. **Section 5** present the limitation of our study, finally **Section 6** present conclusion and future directions.

## 2. Overview

### 2.1 Mobile banking

Mobile banking applications changed financial management by providing users with speed and security together with convenience. Users employ these applications to view their account balances and fund transfers and bill payments while also requesting loans through their mobile devices. ^26^ Owing to technological progress, banking services have become more accessible for individuals located in distant areas, thus eliminating their need to visit bank branches. The security features of banks are enhanced through developments such as biometric authentication and encryption to protect against user risk exposure. ^2^

### 2.2 Biometric authentication

**Figure 2.**
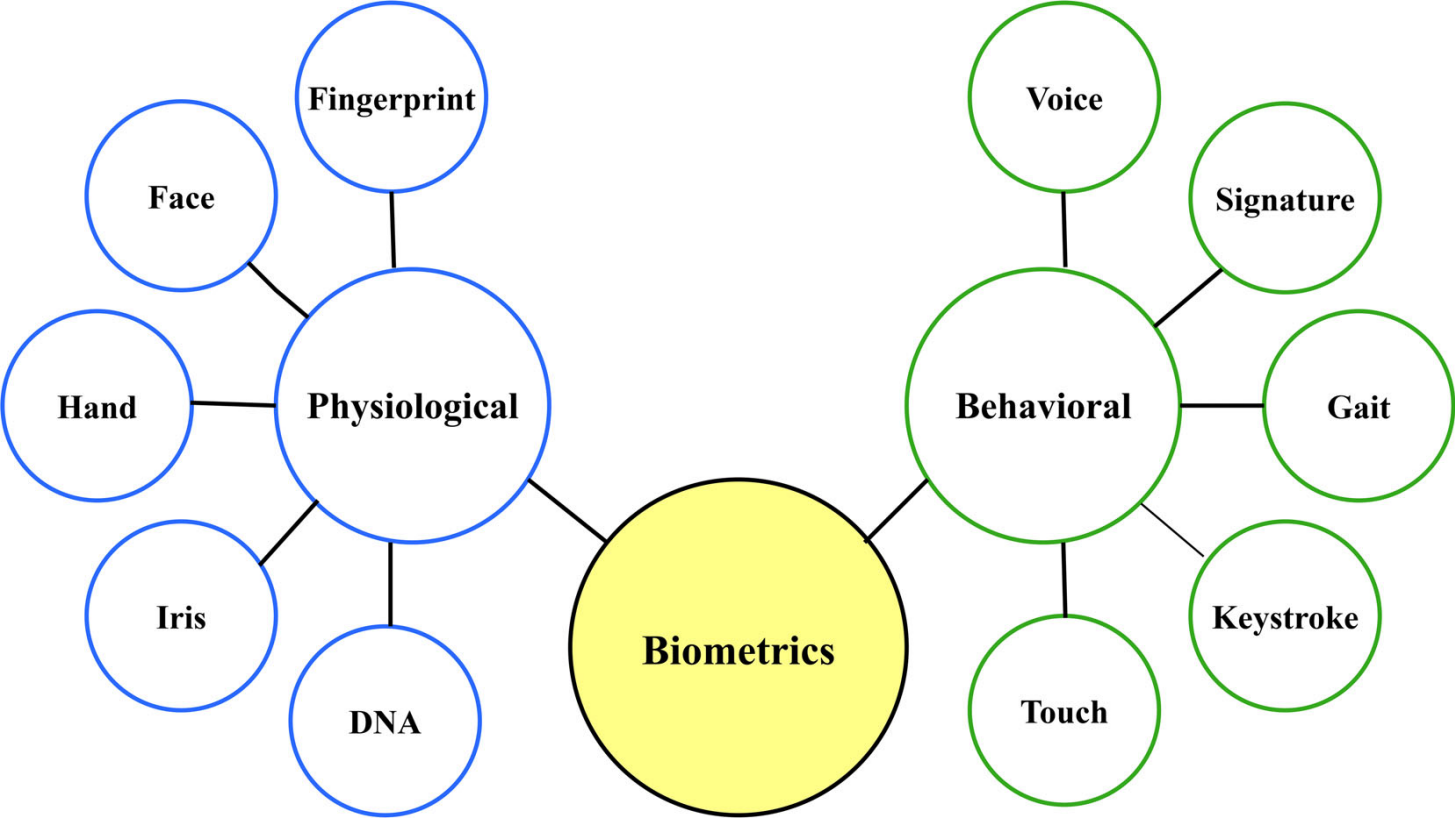
Classification of common biometric authentication.

Biometric authentication has also augmented digital security in that it is stronger and more convenient compared to the use of passwords or PIN-code-based access control systems. As compared to traditional systems, biometric authentication systems also validate identity using individual physiological and behavioral attributes i.e. fingerprints, facial characteristics, the pattern of the iris, voice recognitions, and the dynamics of keystroke ^27^ which are naturally very hard to imitate or steal. ^28, 29^ Biometric authentication continues to gain momentum because mobile banking needs cryptographic protection, as do healthcare services, ^30^ border security systems and enterprise network access systems. Biometric solutions have gained prominence among organizations and financial institutions to build improved security systems because these institutions face escalating cyber risks and data breaches. ^31^ The benefits of the enhanced security and convenience that biometrics provide systems include serious privacy and ethical risks, data safety and security challenges, and system weakness problems. Figure 2 shows the two types of biometric authentication and presents the common methods used.

The security model of user authentication via biometrics has several powerful attributes that establish it as a modern choice to protect digital systems. The main advantage of biometric user authentication lies in its security because individual traits such as fingerprints, facial features and iris patterns are unique and difficult to steal or duplicate from traditional passwords. ^32, 33^ Biometric systems increase user convenience because users do not need to memorize complicated passwords or transport physical security measures. ^34^ The system of biometric authentication contains important weaknesses that need to be addressed. System inaccuracies can produce either unauthorized access or user dissatisfaction due to false positive or false negative results. ^35^ Biometric data cannot be altered after a breach occurs in the way that passwords can be changed, thus resulting in severe privacy and security issues when databases are compromised. ^36^ User consent and data protection statutes generate serious ethical and legal issues that emerge when managing biometric data collection and storage processes. User access, which relies on biometric authentication, should be deployed with appropriate precautions while receiving supplemental security safeguards for data protection. ^37^

Biometrics have their strengths and weaknesses, ^38^ as well as the areas in which they are commonly used. For example, fingerprint and facial recognition work well with mobile phones because the phone already has scanning devices. ^39^ Therefore, they are widely used in various digital systems, such as banking systems, healthcare systems, and other mobile applications. Table 2 provides a brief overview of their primary characteristics such as strong and weak points as well as the common uses in digital environments.

**Table 2.** Analysis of some biometric authentication approaches including descriptions, strengths, weaknesses, and typical use cases.

Biometric method	Description	Strengths	Weaknesses	Common use cases	Ref.
**Fingerprint Recognition**	Scans and matches unique patterns in a user's fingerprint.	Highly accurate, fast, and widely adopted.	It can be affected by dirt or injuries; it requires physical contact.	Smartphones, laptops, access control systems.	^40, 41^
**Facial Recognition**	Analyzes facial features to verify identity.	Contactless, convenient, and fast.	Can be fooled by photos or videos; lighting and angle variations may affect accuracy.	Smartphones, airports, security checkpoints.	^42– 44^
**Iris Recognition**	Scans the unique patterns in the colored ring of the eye.	Extremely accurate and difficult to forge.	Requires specialized hardware; can be intrusive.	High-security facilities, government systems.	^45^
**Voice Recognition**	Analyzes vocal characteristics to verify identity.	Convenient and noninvasive.	It can be affected by background noise or voice changes due to illness.	Call centers, banking, smart home devices.	^46^
**Retina Scanning**	Scans the unique blood vessel patterns in the retina.	Extremely secure and accurate.	Invasive, requires proximity, and expensive hardware.	Military, high-security environments.	^47^
**Hand Geometry**	Measures the shape and size of the hand.	Reliable and easy to use.	Less unique compared to other biometrics, requires physical contact.	Time and attendance systems, access control.	^34^

### 2.3 Role of biometrics in mobile banking

The adoption of mobile banking biometric authentication for purely secure account access has become widespread because of the widespread implementation of modern financial services digital transformation, with improved efficiency and user experience of verification processes. User account passwords combined with PIN-based authentication face growing risks from cyber intruders, who exploit phishing attacks, steal credentials and gain unauthorized system access. ^31, 48^ Online banking security evolves through biometric authentication, which is used physical and behavioral features to provide safe access and protection from fraud. ^28^

Mobile banking security, along with fraud prevention, is one of the fundamental purposes of biometric authentication systems. The security of mobile banking in physical biometrics, such as fingerprint scans, facial verification, hand, iris and vein methods, is important for authenticating authorized users. ^32, 49^

Biometrics offer very high resistance against replication and remain difficult to counterfeit, which protects users from unauthorized transactions as well as identity theft. ^50^ Continuous authentication from behavioral biometrics becomes essential since it analyzes touch interaction patterns and keystroke dynamics along with voice patterns and typing speed, making network breaches more difficult for cybercriminals. ^51^ Biometrics serves as a key instrument for delivering improved accessibility while providing excellent user experience. The user experience becomes more convenient through biometric authentication since users obtain immediate and effortless access to mobile banking applications without needing passwords to remember. Security levels are enhanced through this system because users do not need to manage passwords. Financial inclusion grows stronger through biometric authentication because it enables users who lack literacy skills or disabilities to protect banking services by using their fingerprint or other traits instead of standard user authentication, such as passwords/PINs. ^46^

Biometric authentication users of mobile banking benefit from its advantages while dealing with privacy risks and security vulnerabilities, which include data protection and system protection issues. ^52^ Any unauthorized access to stored biometric information poses substantial risks to users because each person has permanent and distinctive data. To protect biometric data security banks, encryption advances in combination with blockchain-based storage systems and multiple authentication factors have been employed. ^53^

By integrating AI and ML, mobile banking authentication systems can gain the ability to detect more fraudulent transactions alongside the delivery of personalized banking service options to customers. ^54^ The future of mobile banking security is moving toward a safer and more efficient digital financial environment because biometric authentication maintains a balance between security and convenience and privacy. ^55^

### 2.4 Overall rating

The concept of mutual compensation enables authentication security by properly utilizing each authentication variable to eliminate their individual weaknesses ( Table 1). A secure authentication system emerges when users provide PBA, KBA and BBA that protects against multiple types of security attacks. People trust the knowledge authentication type for its familiar design, yet this element remains exposed to password intrusion attacks. ^56^ When ownership requirements for physical tokens or devices are applied together with passwords, the system provides enhanced security even when a password becomes exposed. ^57^ The cost and risk exposure for the ownership element arises from losses connected to token or device disappearance or theft. Security measures benefit from the biometric element because it brings both security through uniqueness and convenience while belonging to the user. Strong protection of biometric data is essential to minimize password-related attacks while physical tokens remain necessary, yet the implementation leads to either positive or negative false negative outcomes. ^58^

Although the three authentication categories (KBA, PBA, and BBA) ^16^ each offer specific advantages, they leave gaps when used alone that can be exploited. KBA is simple but has the lowest security level in terms of phishing and brute-force attacks; PBA is a strong security mechanism based on use of one-time codes or tokens, but it is prone to device-loss attacks; BBA is a strong identity binding mechanism but raises privacy and hardware-cost issues. Thus, our analysis supports a hybrid multi-factor approach whereby complementary factors compensate for the weakness of each other. In environments that require high security, like mobile banking, a three-factor setup (password + token + biometric) offers the best balance of protecting against the theft and spoofing of credentials. ^13^ However, in lower risk or resource constrained environments a two-factor scheme (i.e. PIN + OTP) may provide sufficient security with better usability. This aligns with recent banking practices summarized in Table 7, where most institutions adopt mixed MFA frameworks combining knowledge, possession, and inherence factors.

## 3. Methodology

**Figure 3.**
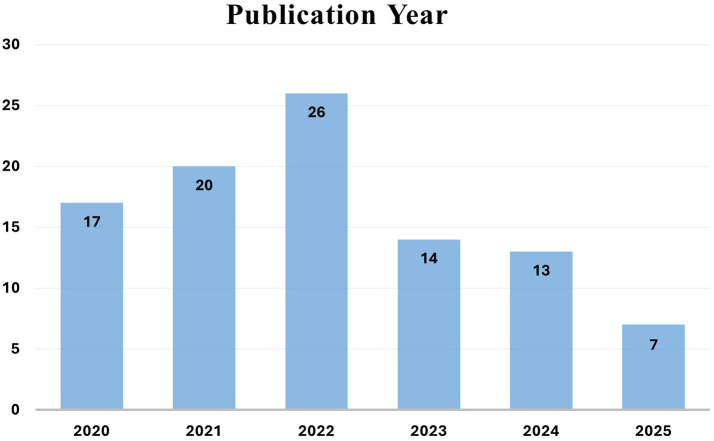
Distribution of the 97 reviewed research papers by publication year (2020-2025), illustrating the growth trend in biometric research for mobile banking.

The Preferred Items for Reporting Systematic Reviews and Meta-Analyses (PRISMA) guidelines were first released more than a decade ago. ^59^ The PRISMA method assists researchers by providing standards for accurate reporting of systematic reviews and meta-analyses. Systematic reviews are considered by decision-makers in areas such as the IoT, computer security, smart homes, supply chains, industries, and other domains as important sources of information that are collected in a systematic and transparent manner. ^60^ Some of the PRISMA items have provided a comprehensive and systematic study of the applications of biometric authentication in the mobile banking sector. ^3^ The literature review in Figure 3 includes the most recent studies related to biometric authentication technology, which are used in mobile banking to increase security. The activities listed below have had a significant effect on the results of systematic surveys. Out of the total 180 references, 97 papers met the inclusion criteria and are analyzed as part of the evidence set. The remaining references are cited to provide general background, definitions, or contextual support but were not included in the systematic synthesis.

### 3.1 Research questions

This research aims to evaluate the biometric authentication methods currently adopted for online mobile banking user access. Also, this research examines the effects of cyber threats on online banking user authentication and presents examples of biometric authentication systems used by major banks worldwide, also examines the advantages, disadvantages, aims and challenges through the following research questions:

1. Which **biometric authentication** methods are currently used in mobile banking systems?
2. What are the main **security threats and vulnerabilities** affecting biometric authentication in mobile banking?
3. How do major banks worldwide **implement and integrate biometric authentication** into their mobile banking applications?
4. What is the key **usability, privacy, and user acceptance challenges** related to biometric authentication in mobile banking?
5. What are the **limitations and future research directions** in improving biometric-based authentication for secure and convenient mobile banking?

### 3.2 Search strategy

A collection of academic papers focused on biometric authentication served as the basis for our review. The selected time span begins on January 1, 2020, and ends on July 1, 2025. This stage involved focused examination of scientific digital libraries and databases alongside searches of keywords and reference management tools and search processes. The next sections delineate the processes described.

### 3.3 Scientific digital libraries

The analysis took place through major English-language scientific digital libraries and databases. Science Direct, Scopus, IEEE and Google Scholar formed the database scope for this SLR.

### 3.4 Search for keywords

**Figure 4.**
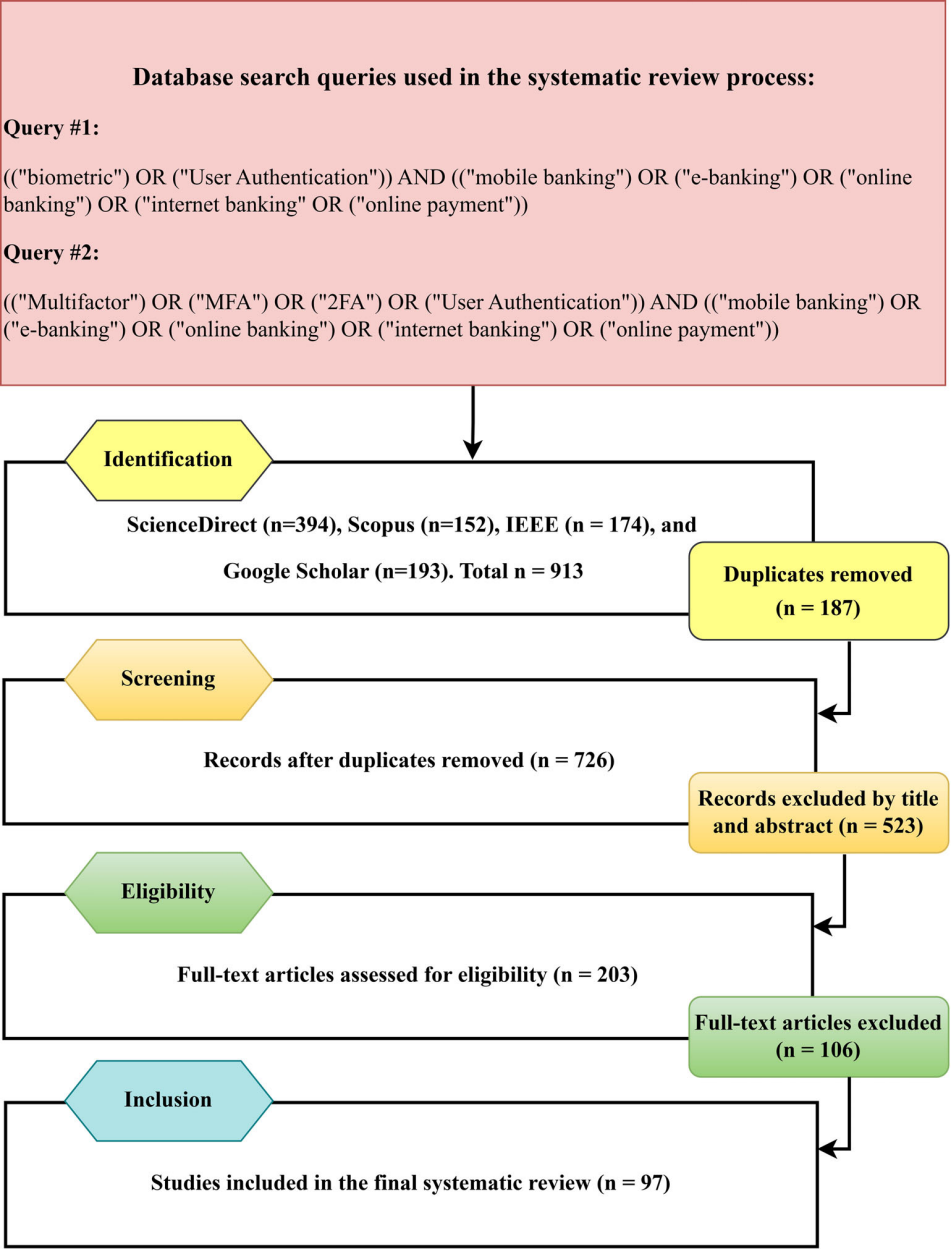
PRISMA flow diagram of the process of selecting and screening studies that were included in this systematic literature review.

The research questions of the SLR served as the foundation for creating the search keywords. The included figure presents alternative search terms. We have also added synonyms and alternatives. The synonym keywords are extracted from the corpus of online banking security related subjects in literature. The search query keywords appear in ( Figure 4) as they were applied to the digital libraries mentioned. To provide comprehensive coverage, the literature search was performed in four major databases: ScienceDirect, Scopus, IEEE, and Google Scholar.

### 3.5 Reference management

The research utilized “Mendeley Reference Manager” v2.132.0 ^61^ to serve as the reference management system for collecting and handling retrieved scientific papers. Using Mendeley Reference Manager v2.132.0 allowed researchers to perform quick document management features on their internal database.

### 3.6 Selection of the study

We examined the research papers that led to the selection of suitable content for our final evaluation using specified inclusion and exclusion criteria. We examined each paper to check its application toward the study’s goals. The review used predefined inclusion criteria to identify and analyze suitable study materials, which led to reliable and valid research outcomes. All the results of this research stem from the number of papers that fulfilled the established criteria permitting their entry into our research.

**3.6.1 Exclusion criteria**

All studies that were not published in the English language were excluded. Additionally, book chapters, reviews, periodical articles, theses and duplicate papers are excluded.

**3.6.2 Inclusion criteria**

The inclusion criterion was that the studies were published in English. Only journal publications and conferences that publish studies were included. The biometric authentication in mobile banking research studies covers the methods used in mobile banking, threats, strengths and weaknesses, as well as the methods used in major international banks and user usability challenges and limitations.

**3.6.3 Results**

The research process resulted in 97 articles through the elimination of duplicate and unrelated studies. The preferred items for reporting systematic reviews and meta-analyses. Two complementary search queries were applied to ensure coverage of biometric authentication keywords ( Figure 4).

The screening process based on PRISMA is illustrated in Figure 4. A total of 913 records were identified in the four databases (Science Direct = 394, Scopus = 152, IEEE = 174, Google Scholar = 193). After elimination of 187 duplicates, 726 unique papers were screened. Following title and abstract screening, 523 papers were excluded, and 203 full-text articles were eligible for further screening. Based on the inclusion and exclusion criteria, 106 papers were deemed ineligible, and 97 studies were finally included in this systematic review.

## 4. Analysis and Discussion

This section presents various data samples drawn from relevant studies and provides an evaluation and interpretation of the SLR findings.

### 4.1 Biometric authentication methods used in mobile banking

**Figure 5.**
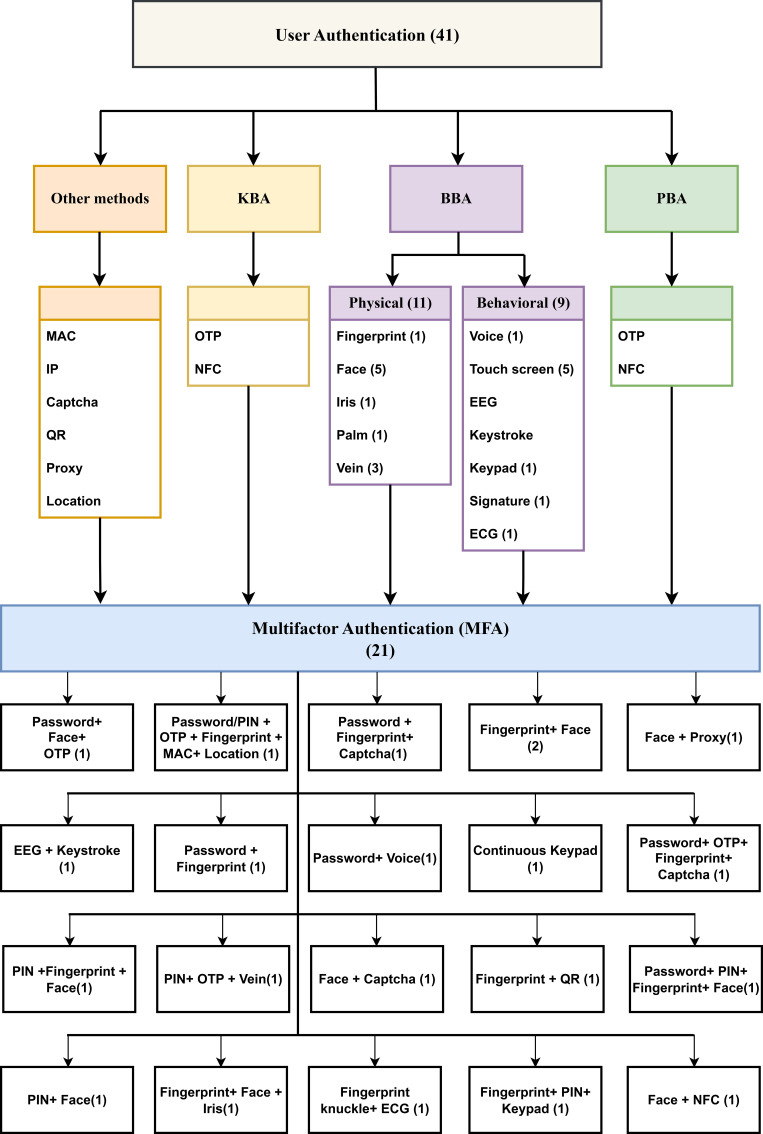
Biometric authentication taxonomy in internet banking, methods based on knowledge, possession and biometrics factors.

This section provides an answer to research **RQ1**: “Which **biometric authentication** methods are currently used in mobile banking systems?”. The gathered literature appears in Table 3 for 41 studies that are analyzed in three parts: description, year and reference. The taxonomy in Figure 5 illustrates different online payment biometric approaches, which are classified as BBA and MFA. The taxonomy structure enables a complete comprehension of the different authentication approaches, which demonstrate that all current methods operating in online banking use biometrics.

**Table 3.** Summary of sample data of the chosen studies (2020-2025) to 41 study, outlining each of the biometric authentication methods, its aims, and originated sources.

Year	Ref.	Description
2020	^52^	A MFA for the Smart Online Banking System (SOBS) uses face recognition authentication (FRA) or biometric fingerprint authentication (BFA) with digital signatures are proposed to enable bank customers to complete transactions.
	^62^	This paper proposed a novel approach based on deep neural network to extract facial features.
	^46^	This research aims to enhance security authentication based on voice recognition, can be utilized for speaker identification, regardless of the language being spoken.
	^50^	This research introduces a secure biometric online banking system which uses three-factor authentication to evaluate service requests through banking portals. These factors are (Password, random the system shows images to users who need to select three familiar images within the interface images and fingerprints).
	^17^	The authors propose an authentication model for securing mobile banking applications based on hand-based biometric authentication.
	^64^	Online banking authentication gets a supervised Machine Learning-based framework from the authors who developed it for continuous behavioral biometric user identification. This framework represents an improved variation of the “Biotouch” technology for touch dynamics identification.
	^68^	The paper introduces a biometric authentication system that uses two methods combining Biometric technology with proximity sensors to provide secure robust and flexible authentication. Biometric fingerprint identification security techniques unite with shuffling keypad methods to boost the security strength in Automated Teller Machines (ATM) operations.
	^63^	A new authentication system uses contactless vascular biometrics to recognize wrist veins as part of the modality system.
2021	^69^	This paper proposed an application for online banking for overcoming the vulnerabilities present in current online banking applications. This application based on facial recognition and proxy detection including “tripleDES” encryption to enhance the security of the work.
	^28^	This paper presents framework based on mobile screen swipes and touch data as a possible verification method for user authentication in mobile banking.
	^70^	The authors presented an authentication model using Fingerprint scanning biometric to provide access to ATM machine.
	^71^	This paper introduces a novel approach to anti-spoofing third-factor authentication method for (ATMs) which uses behavioral-based biometrics Keypad Typing Rhythm Identifier (KTRID).
	^72^	In this paper, the authors present two-level combined authentication method (2 L-IAM). At the first level, the end user login to their online Banking port using either PIN or Fingerprint Matching (FPM). At the second level, end users are authenticated by face recognition (FR) should they initiate a transaction classified as sensitive.
	^73^	This study incorporates user biometrics based on either fingerprint or facial recognition obtain and verify data from the Internet of Things (IoT) device through bank-registered authentication methods which include IP address tracking and digital certificates.
	^25^	This research develops a framework using Elliptical Curve Cryptography (ECC) within Virtual Private Network (VPN) security for performing safe financial operations through MFA using password and voice recognition based on both authentication codes and biometric identification systems.
	^74^	The DAKOTA framework proposes mobile banking security improvement through behavioral biometrics authentication methods based on sensor and touch screen-based continuous authentication. Touch screen data and motion sensor data serve distinct roles to increase application security.
2022	^11^	This paper proposes a new authentication framework for detecting FingerVein (FV) is formed by the work for safe authorization utilizing Enhanced Sigmoid Reweighted based Convolutional Neural Network (ES- “RwCNN”).
	^51^	This paper proposed continuous authentication on mobile devices incorporate touchscreen–swipe interactions without limit as well as keyboard input timing patterns.
	^75^	A novel approach to face anti-spoofing introduces a modified combination of differences of Gaussian (DOG) and angle-difference-ternary correlation-pattern (ADTCP) descriptors.
	^32^	This paper demonstrates an authentication framework which employs novel pupil segmentation through a combination of multiscale gray-level co-occurrence matrix (MSGLCM) with multirange circle Hough transform (MRRCHT). The pupil texture extraction proceeds accurately when using this segmentation method followed by Hough transform application to the outer Iris region.
	^76^	The authors proposed a multimodal Self-ONN based on Raw Electroencephalogram (EEG) and keystroke data.
	^77^	The research proposed an unavoidable authentication approach through mobile device fingerprinting-based identifier and authenticator for mobile banking applications (MDFIA). MDFIA functions as the name for this authentication system.
	^78^	This paper proposed a complete solution to enhancement atm security and privacy by using facial authentication technique.
	^79^	In this paper, the authors developed an application for online system authentication like mobile banking based on face recognition and text extraction.
	^80^	The paper proposes a concept of using a person's vein pattern and OTP/PIN as a method of contactless authentication. It is an extremely safe verification procedure because no two people in the world, not even identical twins, can have the same palm vein structure or pattern. Additionally, it is more secure because it is nearly impossible to replicate the palm vein pattern.
	^49^	The authors proposed a web-based application authentication system for bank employees using passwords, fingerprints and OTP.
	^81^	The article presented a new real-time contactless palm vein recognition system MPSNet specifically developed for smartphones with red, green and blue image functionality. A standard back camera with an LED flashlight installed in smartphones enables the system to both detect and identify palm images.
	^82^	This paper introduces an authentication method with passwordless which includes smartphone-based face recognition and Bluetooth-Near Field Communication technology. The system functions through real-time face biometric authentication and secure NFC token transfer as well as Bluetooth detection of device connection for robust anti-phishing and anti-spoofing security that does not need passwords.
2023	^83^	The article establishes a user authentication methodology which utilizes sensor measurements from smartphone devices along with multiple behavioral patterns along with machine learning strategies to address the identified issues. The proposed approach uses device touchscreen combined with motion sensors to obtain behavioral biometric data.
	^84^	This paper proposes a new framework for continuous authentication for smartphones based on behavioral-based biometric by utilizing for user interaction on touchscreen.
	^30^	The proposed method in this paper develops a secure virtual smart card through digital encryption techniques with biometric verification (using Fingerprint) and a QR code and passwordless capabilities enabling safe access to healthcare systems and e-banking.
	^85^	The study established a multilayer 5FA system that selected Password/PIN together with OTP and Fingerprint along with Media Access Control (MAC) Address and Time-Based location to create a stable security solution for online banking.
	^86^	Authors propose a framework based on dynamic signatures for authentication which called: a “Cloud-based mobile biometric authentication framework (BAMCloud)”.
2024	^13^	This paper proposed a MFA for securing mobile banking system that combines passwords, Face recognition and OTP to verify users.
	^87^	This paper proposed a new framework of mobile payment for user verification depending on face ID recognition based on deep learning.
	^88^	This paper presents an android mobile banking application development. The application implements facial recognition technology together with PIN based templates through the Grassmann algorithm approach. The system becomes accessible for users to perform banking operations only after completing two authentication steps.
2025	^89^	The authors developed an authentication system based on processing ECG (electrocardiogram) signals on mobile devices to achieve high levels of accuracy. The process uses distinct qualities of ECG signals to deliver safe mobile device authentication which demonstrates that biometric authentication can boost security protocols.
	^90^	A new deep learning framework described in this paper connects three different biometric modes through electrocardiogram (ECG) with fingerprint features and finger knuckle print (FKP). The combined application of these methods enables an authentication system that reaches high levels of security and efficiency for banking and healthcare applications and higher-security applications.
	^91^	In this project a machine learning authentication system will be developed to protect online voting using facial and fingerprint recognition as security measures for better system protection. The system consists of two fundamental elements which include both the machine learning authentication mechanism and web-based voting platform.
	^92^	This study proposes a new framework based on MFA which combines three types of fusion technique (feature-level, score-level, and decision level) integrated into three types of biometrics modalities (fingerprint, facial recognition, and iris).
	^93^	This paper presents a hybrid face biometric authentication that integrates the strength of deep learning specifically CNN and (ResNet) with Local Binary Pattern (LBP) method.

**4.1.1 Biometric authentication methods**

The BBA category uses two types of biometrics, including physical traits, which consist of fingerprints, facial IDs, irises, hands and veins, ^11, 17, 32, 62, 63^ alongside behavioral traits, which include voice patterns, ^46^ “biotuch” (dynamic/continuous touch authentication), ^28, 64^ and tapping behavior. The other behavioral biometric trait was implemented by, ^65^ who proposed a new activity recognition model for smartphone applications based on physical activities that are detected by collecting data from different sources, such as biometric sensors or body-worn sensors. The authentication system uses unique biological traits from users to create a high level of security protection. The authentication approach of behavioral biometrics shows great reliability in authorization but creates problems involving data protection and storage security requirements. Organizations use biometric information in addition to developing preventative solutions to combat unapproved system access and illegal information handling. ^66^ Various users sometimes encounter difficulties when organizations attempt to establish biometric authentication systems. Online banking system implementations of biometric technology require thorough consideration of hardware needs together with quality standards and acceptance levels from users. ^67^ Table 3 provides a description and year for each study.

**4.1.2 Integration of biometrics within MFA**

Any authentication system built with a single authentication factor remains vulnerable to security threats regardless of the use of fingerprint scans, facial recognition, palm recognition, passwords or PIN. Authentication system developers develop their systems via integrated MFA. ^94^ The integration of BBA methods, including fingerprints, faces, irises, hands, veins and behavioral biometrics, serves as the main authentication component for the MFA and 2FA systems. ^95^ PBA authentication methods consist of OTPs combined with tokens and NFC ^96^ and operate when enhanced with other supporting factors. Various security elements, such as proxies, QR codes, geolocation, IP address MAC addresses and “CAPTCHA,” have joined the MFA and 2FA approaches to increase security measures. Multiple authentication methods used together by banks create enhanced system security, which implements multiple defense barriers for attackers to break. The security system becomes protected by multiple layers if attackers gain access to one part because the remaining layers shield the system from further attacks. ^97, 98^ Users must first type their password before submitting their fingerprint for authorization purposes during system access. This authentication logic increases the security level by making it difficult for attackers to compromise the system despite knowing the user password. ^99^ Online banking can establish a secure and all-encompassing authentication system through 2FA and MFA, which reduces the threats caused by depending on a single authentication mechanism. The analysis of previously discussed online banking authentication approaches that can be combined with biometric factors yields the results in Table 5, which presents their strengths together with weaknesses. Based on 41 studies, this research examines the use of biometric authentication methods in online mobile banking, including their role within MFA. These methods are presented in Table 4 and Figure 5. Table 4 provides a comprehensive list of these authentication strategies, complete with their corresponding references.

**Table 4.** Overview of biometric integrated within the MFA techniques used in the studies reviewed with modalities used and references provided.

Authentication Method	Password	PIN	OTP	Fingerprint	Face	Iris	Palm/Hand	Vein	Voice	Continuous Authentication	Signature	MAC	Location	Captcha	Proxy	QR	EEG	ECG	Keystroke	Keypad	IP Address	FKP	NFC
Ref.																							
^52^				**O**	**O**																		
^62^					**O**																		
^46^									**O**														
^64^										**O**													
^11^								**O**															
^13^	**O**		**O**		**O**																		
^86^											**O**												
^28^										**O**													
^70^				**O**																			
^72^		**O**		**O**	**O**																		
^25^	**O**								**O**														
^73^	**O**	**O**		**O**	**O**																**O**		
^51^										**O**										**O**			
^74^										**O**													
^17^							**O**																
^87^					**O**																		
^85^	**O**	**O**	**O**	**O**								**O**	**O**										
^50^	**O**			**O**										**O**									
^69^					**O**										**O**								
^30^				**O**												**O**							
^71^																				**O**			
^84^										**O**													
^75^					**O**																		
^32^						**O**																	
^76^																	**O**		**O**				
^83^										**O**													
^77^	**O**			**O**																			
^78^					**O**																		
^79^					**O**									**O**									
^80^		**O**	**O**					**O**															
^49^	**O**		**O**	**O**										**O**									
^88^		**O**			**O**																		
^89^																		**O**					
^90^				**O**														**O**				**O**	
^91^				**O**	**O**																		
^92^				**O**	**O**	**O**																	
^93^					**O**																		
^81^								**O**															
^68^		**O**		**O**																**O**			
^63^								**O**															
^82^					**O**																		**O**

Figure 5 presents a detailed taxonomy scheme of authentication methods that were specifically developed to protect online banking systems. The taxonomy system groups authentication methods into four basic categories, including the PBA, BBA and KBA approaches and MFA methods. The BBA is divided into two parts: physical biometrics, including fingerprints, faces, irises, hands/pales and veins, and behavioral traits, including voices, touch screens, EEGs, keypads, signatures and ECGs. The KBA consists of a password PIN. The PBA includes OTP and NFC. Other types of authentications include MAC address, IP address, CAPTCHA, QR, proxy and location. The MFA introduced a good security solution by combining the 2FA or MFA from the previously mentioned methods, which illustrates the wide range of security methods used for user verification. This research focuses heavily on biometric authentication by providing detailed information about fingerprints, facial recognition, irises, palm, voice recognition methods, touchscreens, and keystroke dynamics as behavioral verification indicators. This taxonomy explains MFA, which requires users to combine different authentication components such as passwords together with OTP or fingerprints in combination with the MAC address to increase security. The taxonomy establishes itself as a beneficial reference for recognizing the complex authentication methods that modern digital finance systems implement.

**Figure 6.**
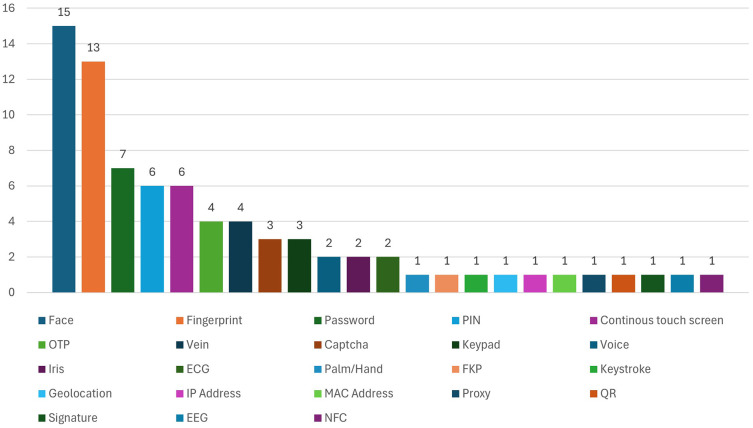
Frequency distribution of authentication techniques used in biometric systems among studied mobile banking studies.

Among all the biometric authentication methods face, fingerprint, password, PIN and continuous touch screen authentication methods are the most used since they appear 15, 13, 7, 6 and 6 times, respectively, in total. OTPs and veins appeared 4 times each, and voice was used only 2 times throughout the given data and iris. The authentication system uses several types of verification, including CAPATCHA and keypads, which appear in 3 cases each. One mention exists for each of these authentication methods: palm, keystroke, time-based location, MAC address, IP address, proxy, QR, signature, EEG, ECG, FKP and NFC. MFA security becomes more effective because face and fingerprint detection are used multiple times with additional KBA, such as password/PIN and OTP. Multiple security layers protect sensitive online banking data since MFA operates together with 2FA by employing several authentication techniques. Figure 6 presents the frequency of authentication methods in biometric authentication methods contexts that are implemented in the banking sector.

This study presents a comprehensive and well-structured systematic literature review on biometric in online mobile banking systems, which offers valuable taxonomy, security threat analysis, and insights into global banking practices. Its key achievement is to synthesize the recent research (2020-2025) and provide a clear classification of authentication methods and challenges. So, the absence of quantitative performance and empirical validation comparisons limits its practical depth. Overall, the study provides a strong theoretical foundation and serves as a reliable reference for both academia and industry.

**4.1.3 Evaluation of the methods of mobile banking**

Table 5 analyzes different authentication methods from our study based on their strengths and weaknesses and corresponding research sources. Simple security systems based on passwords and PINs continue to be popular because they are quick to set up while attackers take advantage of vulnerabilities that result in brute-force attacks and phishing schemes as well as shoulder surfing. User authentication through PIN combined with facial recognition delivers better usability while providing touchless access at the cost of reduced security effectiveness. The implementation of passwords, fingerprints, faces, OTP and locations coupled with MAC address recognition increases security, whereas the use of external communications remains a weak point, and spoofing presents a persistent threat. Biometric authentication through fingerprint scans mixed with facial identification combined with iris analysis creates secure user authentication solutions despite their limitations in terms of external conditions and the need for specific hardware systems. Emerging techniques such as EEG and touchscreen behavior offer unique behavioral or physiological markers but face limitations in practicality and consistency. MFA represents a powerful security method that achieves adequate protection from MFA, while users experience decreased convenience and dependence on devices during authentication processes. Different digital systems require customized authentication strategies that depend on their unique operational needs according to this analysis.

**Table 5.** Comparison of the authentication strategies employed in mobile banking (2020-2025), with their key strengths and weaknesses.

Method	Strengths	Weaknesses	Reference
**Password**	Simple to implement and widely used	Susceptible to brute-force, phishing, reuse attacks	^60, 100, 101^
**PIN**	Quick, minimal memory load	Easy to observe (shoulder surfing); often reused	^101^
**Graphical Password**	More memorable; harder to guess	Usability issues; vulnerable to observation	^102, 103^
**QR Code**	Fast, contactless authentication	Can be used for phishing; requires camera	^104^
**OTP**	Temporary and time-bound; improves security	Phish able; reliant on delivery medium (SMS/email)	^101, 105^
**MAC Address**	Device specific; useful in background authentication	Spoof able; not user-unique	^3, 106^
**CAPTCHA**	Prevents bots; low cost	Poor usability; accessibility issues	^107^
**Proxy Detection**	Identifies IP masking attempts	Can yield false positives; circumventable	^108^
**Geolocation**	Provides context-aware authentication	Spoof able; raises privacy concerns	^109^
**Signature Verification**	Familiar; good for legal systems	Inconsistent; easy to forge	^110^
**NFC**	Fast, contactless, widely used in mobile payments	Limited range; vulnerable to relay attacks	^111^
**2FA/MFA**	High security combines multiple factors	Less convenient; requires multiple devices or tokens	^3^
**Fingerprint**	Convenient; widely supported in devices	Not reliable when wet/damaged; spoof able	^49^
**Face ID**	Touchless; user-friendly	Can be spoofed by photos or masks; lighting affects accuracy	^12^
**Iris Scan**	Highly accurate and unique	Expensive hardware; less user-friendly	^112^
**Palm Recognition**	High accuracy; touchless	Requires specialized scanners	^17^
**Voice Recognition**	No touch input; good for phone auth	Affected by background noise and illness	^46^
**Vein Pattern**	Internal biometric; hard to replicate	Requires IR scanners; costly	^80^
**EEG (Brainwave)**	Highly secure and unique	Impractical; requires specialized equipment	^76^
**Keystroke**	No additional hardware needed	Inconsistent due to emotional state or fatigue	^2^
**Touchscreen (swipe, tap, scroll)**	Continuous passive auth; behavioral uniqueness	Variability in usage, mood, and devices	^64^

The results summarized in Table 5 emphasize that each category of authentication provides different benefits and weaknesses and confirms the fact that there is no one method which provides total protection against the wide range of cyber threats in the mobile banking environment. Instead, a combined optimization of security strength, usability and implementation cost is required to achieve practical and scalable protection. KBA methods, including passwords and PINs, are the most common because they are so simple and easy to implement. However, they are also the weakest layer of defense, because they are highly vulnerable against brute-force, phishing, and credentials-reuse attacks. ^3, 100, 101^ Moreover, users often use predictable passwords or reuse the same passwords for different platforms, which makes compromise much easier. PINs are faster to enter, and they have very little cognitive load, but they are short and vulnerable to observation (shoulder surfing) which limits their effectiveness on their own.

PBA methods such as OTP, NFC-based tokens provide stronger protection with an introduction of time factor or hardware-binding factor. OTPs greatly decrease the chances of the credential replay but rely on the reliability of the communication and can be decrypted on their way or stolen via a SIM-swap attack. NFC cards and hardware tokens provide a physical guarantee although they involve the user carrying or maintaining an external tool, increasing usability and logistical costs. ^111^

BBA methods with reference to the fingerprint and facial recognition processes provide an ideal compromise between security and convenience, that is why they quickly become standard in any large mobile banking application. ^12, 49^ Fingerprint recognition is more accurate and faster to verify, and facial recognition can be used contactless and more conveniently. However, they are vulnerable to the quality of sensors, lighting, and spoofing as well as cause serious privacy issues because biometric identifiers are not reversible once compromised.

More expensive modalities, like iris, vein or palm recognition, are very resistant to anti-spoofing and very accurate, but expensive specialized sensors preclude their mass-market use. For example, routine account access may rely upon simple 2FA such as PIN + OTP and high value or cross-border transactions may require full MFA including a biometric check. This strategy offers both convenience and high level of security.

Moreover, incorporating AI-based behavioral monitoring into the MFA pipelines will enable a continuous risk evaluation process and allow making real-time adjustments without undermining the usability. Generally, the comparative synthesis implies that the most resilient way of authentication of mobile banking is hybrid, context-aware and adaptive MFA architectures. By combining the desirable attributes of the various factors such as password familiarity, token possession and biometric factors, but at the same time balancing the weaknesses of these factors, it will be possible to ensure high assurance and acceptance by the user for banks. The findings of this comparative evaluation reflect contemporary trends in international banking practice, in which the most important institutions are increasingly using biometric and MFA in tandem to enhance resilience against changing cyber threats. ^113, 114^

Although the heterogeneity of the reviewed studies made it impossible to conduct a complete quantitative meta-analysis, a systematic summary of the reported performance ranges was built to support the quantitative patterns and trends of biometric modalities deployed on mobile devices. The summarized values of false acceptance rate (FAR), false rejection rate (FRR), and equal error rate (EER) - and indication whether liveness detection and spoofing resistance mechanisms were used - are shown in Table 6, which is for studies published between 2020 and 2025.

**Table 6.** Summary of reported biometric and multimodal authentication systems in recent literature, highlighting their typical datasets or devices, reported EER/FAR/FRR rates, accuracy, presence of liveness detection, and spoofing resistance levels.

Typical dataset/Device	EER / FAR/FRR (%)	Accuracy %	Liveness detection	Spoofing resistance	Ref.
Prototype survey (170 users, Brunei)	— / — / —	N/A	N/A	Medium	^52^
CAS-PEAL DB (99k images, 1040 users)	— / — / —	98.52	N/A	N/A	^62^
Custom dataset (23 users), SecuGen scanner	— / — / —	N/A	N/A	N/A	^50^
HKPU 3D/2D Hand DB (570 samples)	— / — / —	94.7–100	N/A	Moderate	^17^
Custom app (51 users, Android sensors)	1.88–9.85/ — / —	82.5–98.2	N/A	Moderate–High	^64^
MFS100 Sensor, Simulated ATM	— / — / —	100	Yes (Basic)	High	^68^
UC3M-CV2 (Smartphone NIR camera)	6.82–18.7/ — / —	N/A	N/A	Partial (No PAD)	^63^
Custom Dataset (20 img/user, smartphone)	— / — / —	97	Yes (Proxy detection)	High	^69^
Prototype Web Portal + Survey (n = 170)	— / — / —	N/A	N/A	Moderate	^72^
IoT Prototype (Design Science)	— / — / —	N/A	N/A	High	^73^
Simulated Cloud Banking Prototype (VPN)	— / — / —	N/A	Partial (Inherent)	Strong	^25^
Custom dataset (45 users, Samsung/Xiaomi)	3.5/ — / —	~99	N/A	High	^74^
N/A	— / —/2.35	97.05	Yes (Inherent)	High	^11^
13 user datasets (Nexus 7 tablets)	~0.1/ — / —	Up to 100	Yes (Implicit)	High	^51^
NUAA, MSU-MFSD, Replay-Attack DBs	1.57/ — / —	99.03	Yes (Texture-based)	High	^75^
CASIA v4, MMU V2	0.44/ — / —	87.7–94.5	Yes (Implicit)	High	^32^
Custom ATM dataset (Raspberry Pi)	— / — / —	≈82–85	Yes (Real-time)	Low–Moderate	^78^
Real user documents (Web portal)	— / — / —	N/A	Partial (Live capture)	Moderate–High	^79^
NIR Scanner Prototype (Raspberry Pi)	— / — / —	High (≥98)	Yes (Contactless)	High	^80^
Custom Android app (15 users)	— / — / —	≈90	N/A	Moderate	^49^
Smartphone RGB cameras (NTUST, XJTU DBs)	0.49/ — / —	≈99	Yes (Live capture)	Very High	^81^
Android (BLE/NFC); MobileFaceNet (MS-Celeb DB)	— / — / —	N/A	Yes (Real-time)	High	^82^
HMOG dataset (100 users, Galaxy S4)	3.35/—/ —	98.75% (F1)	Yes (Implicit)	High	^83^
Serwadda et al. dataset (Smartphones)	0.179/ — /—	89	N/A	Moderate–High	^84^
Custom mobile app (IoT/Healthcare)	— / — /—	N/A	Yes (Mutual auth.)	High	^30^
Custom mobile/web prototype (Firebase)	— / — / —	N/A	Yes (Context-aware)	High	^85^
Custom dataset (Smartphones/Tablets)	0.24/ — / —	96.23%	Yes (Dynamic)	High	^86^
Prototype app (Android + Firebase)	— / — / —	N/A	Yes (Context-aware)	High	^13^
AR, MUCT DBs + mobile frames	— / — / —	99.85	Yes (Real-time)	High	^87^
Custom Android app (Razorpay API)	— / — /0.28	≈97–98	Yes (Real-time)	Moderate–High	^88^
ECG-ID, Heartprint, Custom BMD101 DBs	5.61/ — / —	94.39	Yes (Inherent)	Very High	^89^
Synthetic DBs (PhysioNet, SOCOFing, IIT)	0.20 / 0.20/0.21	99.80	Yes (ECG validation)	Very High	^90^
Custom Kaggle dataset (Web platform)	— / — / —	98.0	N/A	Moderate	^91^
LFW, FVC2004, CASIA, UBIRIS DBs	0.085/ 0.02/0.15	99.47	Yes (DL-based PAD)	Very High	^92^
Custom dataset (not stated)	— / — / —	>98	N/A	Moderate	^93^

As shown in Table 6, most of the reported biometric authentication systems after 2020 have an accuracy rate of greater than 95% and a number of multimodal and deep learning-based approaches have achieved EER values below 1%. However, almost half of the discussed studies failed to report FAR/FRR metrics and only a limited set of studies explicitly used liveness detection or anti-spoofing mechanisms. This suggests that while accuracy has improved significantly from mobile-based biometric systems, issues of presentation attack resistance and real-world scenarios of spoofing remain a major research gap.

### 4.2 Threat-facing mobile banking

Internet banking provides users with efficient convenience but remains at risk from multiple cybersecurity threats, which endanger both financial service integrity and confidentiality as well as system availability. ^115, 116^ The financial sector poses three prominent security threats against banking consumers, which include phishing scams that trick customers to disclose sensitive data as well as malware intrusions stealing control of devices for login interception ^117^ and man-in-the-middle (MiTM) attacks that break communication between customers and banking institutions. Ransomware attacks, ^118^ along with brute force methods and credential stuffing, exploit password weaknesses by targeting ordinary users who repeat their passwords across different accounts. Moreover, social engineering methods ^119^ allow users to surrender their confidential information. Security threats affect both personal users and cause substantial damage to financial institution reputation and operational stability. We provide answers to **RQ2** in this section: “What are the main **security threats and vulnerabilities** affecting biometric authentication in mobile banking?”. These studies include:

**4.2.1 Malware attacks**

Mobile banking malware attacks have become increasingly dangerous because smartphone users continually increase their banking activities on mobile devices. The mobile-specific malware category includes banking Trojans and fake banking applications, which lead to theft of login data while also intercepting SMS authentication codes through screen overlay tactics. ^120^ The threats take advantage of users downloading harmful applications together with deceitful links that imitate genuine banking platforms. The protection of mobile banking needs strong security measures and app store monitoring while offering user education programs since mobile users demonstrate limited security awareness and malware persists in adapting. ^100, 121^

**4.2.2 Man-in-the-Middle (MiTM) attacks**

The (MiTM) attack allows cybercriminals to intercept communications between two parties while they cannot detect the security threat. Internet senders become vulnerable to cybercriminals through MiTM attacks, as attackers intercept their sent sensitive information, including login credentials and transaction details, during transmission. Online attackers take advantage of unsecured networks particularly well to launch attacks against public Wi-Fi networks because these networks present communication opportunities. The combination of SSL/TLS encryption protocols with safe programming practices and user training about risk networks makes sensitive banking information secure during online financial activities. ^56, 122, 123^

**4.2.3 Replay attack**

An attacker generates fraud by intercepting authentic transmission data to fool an intended recipient into taking actions that might include transaction authorization. The fraudulent method of playing replays allows attackers to pose as victims and redirect transaction details to a bank for resulting payments or account transfers. ^124^ Attackers exploit old communication protocols without session state validation and timestamping, which they leverage for their attacks. All submitted transactions must pass through time-based authentication protocols, whereas financial institutions need to use nonce-secured requests with session tokens for attack prevention. ^56, 125, 126^

**4.2.4 Phishing attacks**

Criminal online attackers conduct phishing operations for social engineering by pretending to be trusted parties to steal sensitive data that includes usernames with passwords and credit card details. Criminals execute phishing attacks in online banking through fake websites or emails, which are intended to be actual banking sites because they trick users to provide login details and sensitive financial information. Phishing attacks succeed by targeting human mistakes combined with trust to obtain sensitive information, which requires users to develop heightened awareness to stop them. ^121, 127^

**4.2.5 Social engineering**

Attackers use social engineering as a method to force users to reveal sensitive data or perform actions that endanger their banking account security through online interfaces. Attackers use deception techniques such as urgency fabrication along with impersonation and trust-building tactics to trick users. ^57, 128, 129^

**4.2.6 SQL injection**

The attackers conduct SQL injection attacks by adding harmful code to the data strings before they reach the SQL database for processing. SQL injection allows hackers to obtain user credentials, including usernames and passwords. The stolen credentials serve as keys to unauthorized access of user accounts. ^14, 130, 131^

**4.2.7 Keylogger**

A keylogger functions as malicious software that tracks computer keystrokes. A keylogger logs every keystroke typed on a system that has been compromised and captures sensitive data, including passwords, usernames, etc. Your computer can acquire keyloggers through a few methods, including opening tainted attachments and clicking on harmful links together with downloading files from unverified sources. A keylogger that uses the system to record keystrokes becomes operational after installation on a computer. ^29^

**4.2.8 Weak password**

Weak passwords pose security threats to online banking since they provide easy opportunities for attackers to guess them. The entry of unauthorized actors becomes easier when passwords are weak because they gain instant access to sensitive data and user accounts and execute fraudulent schemes. ^123, 132^

**4.2.9 Denial-of-Service (DoS) attacks**

DoS attacks block access for genuine users in online banking operations through a combination of abusive network traffic that saturates system resources. Service interruptions alongside customer dissatisfaction result from this situation, which leads to financial losses for the bank. Some attacks that initiate denial-of-service conditions serve as shields for other more dangerous threats, including data theft. ^98, 101^

**4.2.10 Session hijacking**

Unauthorized session access, which enables an attacker to exploit user identity for online bank account access, is known as “session hijacking.” Network connection interceptions, along with exploiting system vulnerabilities, enable attackers to conduct unlawful activities while acquiring private information. ^133^

The attacks mentioned earlier show vulnerability to the supply chain and third-party or endpoint access of online banking systems, which results in supply chain attacks that involve attackers using external third-party software or vendors to access systems. The security vulnerabilities in banking systems enable hackers to break online banking security, which might result in unauthorized access and data breaches. Attackers execute endpoint attacks by focusing on both the user’s devices and end points. The attackers want to infiltrate the user’s device to access confidential banking data, which results in both financial fraud and unauthorized transactions. ^126^

Financial institutions need to use the security measures presented in Table 7 to build protective online banking systems that secure user financial details. Financial institutions establish better online banking security through a complete strategy that integrates technical solutions such as firewalls and antiviruses, educates users about risks and develops essential policies plus continuous observations of systems. Table 7 summarizes these threats while presenting countermeasures that could serve to prevent them.

**Table 7.** Online banking user authentication threats with potential controls.

Attacks	Potential controls	Reference
**Malware Attacks**	• Antivirus & anti-malware software. • Regular system updates. • Application whitelisting. • User education & sandboxing.	^31, 124, 134^
**Man-in-the-Middle (MitM) Attacks**	• End-to-end encryption (e.g., TLS/SSL). • Certificate pinning. • Secure key exchange (e.g., Diffie-Hellman). • Avoid public Wi-Fi or use VPN.	^135, 136^
**Replay Attacks**	• Timestamps & nonce-based protocols. • Secure tokens with expiration. • Mutual authentication.	^125, 132^
**Phishing Attacks**	• Email filtering (spam/phishing detection). • User awareness training. • Domain monitoring. • MFA to mitigate stolen credentials.	^57, 137, 138^
**Social Engineering**	• Security awareness programs. • Simulated phishing tests. • Clear verification policies. • Insider threat monitoring.	^137, 139, 140^
**SQL Injection**	• Input validation & sanitization. • Use of prepared statements (parameterized. queries). • Web application firewalls (WAF).	^130^
**Keylogger**	• Anti-spyware detection tools. • Behavioral monitoring. • OS hardening & restricted privileges. • On-screen keyboards for sensitive input.	^141^
**Weak Passwords**	• Enforce strong password policies. • Password managers. • MFA/2FA. • Rate limiting and lockout mechanisms.	^142, 143^
**DoS**	• Traffic filtering and rate limiting. • Use of CDNs. • Redundant systems and load balancing. • Anomaly detection (IDS/IPS).	^144^
**Session Hijacking**	• Secure cookie attributes (e.g., Http Only, Secure, Same Site). • Session timeout policies. • Token-based session management. • HTTPS for all traffic.	^133^

### 4.3 Biometric used in international banks

The investigation of **RQ3**: “How do major banks worldwide **implement and integrate biometric authentication** into their mobile banking applications?” takes place in this section. The analysis of authentication practices utilized by international banks provides fundamental knowledge about contemporary changes in online banking safety measures. The selected banks outline their user authentication techniques in Table 8 demonstrates how various popular financial institutions authenticate their users.

**Table 8.** Summary of biometric authentication methods implemented by major global banks, with emphasis put on their security settings and location of operations.

Bank	Country	Authentication methods	Ref.
JPMorgan Chase	USA	Username/password, OTP via SMS/email, biometric (Face ID, Touch ID), device recognition	^25^
Bank of America	USA	Username/password, OTP via SMS/email, biometric login via app (Face/Touch ID), app-based MFA	^3^
HSBC	UK/Global	Secure Key (hardware/token), Mobile Security Key (in-app), biometric login, OTP	^106, 148^
Barclays	UK	PIN sentry device, biometric login, app-based MFA, SMS/email OTP	^149^
Deutsche Bank	Germany	Username/password, mobile TAN (mTAN), photo TAN, biometric login, push notification approval	^150, 151^
BNP Paribas	France	Password + OTP (SMS/email), biometric login, mobile token, app-based confirmation	^152^
Santander	Spain/Global	Password, OTP via SMS/email, Mobile Sign (in-app approval), biometric authentication	^153^
ING	Netherlands	PIN/password, fingerprint/Face ID via app, in-app transaction approval	^114, 154^
Standard Chartered	UK/Asia/Africa	Username/password, OTP via SMS/email, app-based security token, biometric login	^155^
DBS Bank	Singapore	Biometric login, digibank Secure Device, app-based push approval, OTP	^105, 114^
HSBC Hong Kong	Hong Kong	Mobile Security Key, Face ID/Touch ID, OTP via SMS, transaction signing	^154^
RBC (Royal Bank of Canada)	Canada	Username/password, OTP, biometric login, 2FA through Secure Cloud	^155^
NAB (National Australia Bank)	Australia	Password, SMS OTP, biometric login via mobile app, device recognition	^25^

The information in Table 8 reveals the user authentication strategies used by several well-known financial institutions, including JPMorgan Chase, Bank of America, HSBC, Barclays, Deutsche Bank, BNP Paribas, Santander, ING, Standard Chartered, DBS Bank, HSBC Hong Kong, RBC (Royal Bank of Canada), and NAB (National Australia Bank).

Financial institutions utilize MFA systems because they prioritize the defense of customer online account security. Financial institutions mainly employ password authentication as their principal security procedure. Account holders need to build passwords for their financial institutions, which adhere to prescribed guidelines (such as the NIST password policy) that require eight characters or more in length with a mixture of upper and lowercase letters and numbers and symbols. The initial security measure that defends against unapproved system access is the use of passwords. OTP authentication serves as a security approach that numerous financial institutions within the banking sector currently use. New device users must provide a unique OTP password that arrives through SMS or email to finish their login process. Users must provide the received code before their login process can become fully secure. OTP serves only single login sessions and operates with limited validity, making it highly unlikely for attackers to obtain the password. The rising trend in banking shows that physiological biometric identification serves banks as a safer authentication option than passcodes do. Special physical attributes such as fingerprints and facial recognition serve as verification tools to identify customers during their process. ^113^ The authentication method poses challenges to falsify essential data, thus making it an optimal barrier against unauthorized access. Security functions at these banks are implemented via 2FA technology. Users need to enter two security factors along with their password during first-time logins from new devices by providing an OTP or biometric verification. Security protection from 2FA ^145^ creates a double authentication requirement that makes unauthorized access attempts practically impossible. Banks offer alternative authentication solutions for customers, which include USB security keys as well as security questions and challenge/response authentication and voiceprint authentication and device fingerprints. ^146^ Device fingerprints determine the devices used by users, whereas voiceprint authentication depends on a person’s voice specifics. ^147^ A challenge/response system requires the customer to enter authorization codes that originate from bank transmissions to their mobile device. Security keys connected to a USB port work as extended measures for online banking safety by enabling users to enhance transfer constraints through their computer’s USB connector. As customers complete the login process, the system asks them to answer security questions that have already been chosen by the platform, thus activating security authentication. Bank security measures receive ongoing assessment and updates from these banks to maintain timely protection of customer account safety against new security challenges.

We highlighted the use of biometrics in leading banks, extracted from Table 8, to determine the most used authentication methods. Table 9 illustrates the role of the biometric methods used by leading banks.

**Table 9.** Biometric authentication methods implemented in leading banks.

Bank	Biometric technology used	Application area
**JPMorgan Chase**	Facial recognition, palm vein scanning	In-store payment authentication
**Bank of America**	Fingerprint, facial recognition	Mobile banking app
**HSBC**	Fingerprint, facial recognition	Mobile banking app
**Barclays**	Voice recognition	Phone banking services
**Deutsche Bank**	Fingerprint, facial recognition	DB Secure Authenticator app
**BNP Paribas**	Fingerprint	Biometric payment cards
**Santander**	Fingerprint, facial recognition	Mobile banking app
**ING**	Fingerprint	Mobile banking app
**Standard Chartered**	Fingerprint, facial recognition	Mobile banking app
**DBS Bank**	Fingerprint, facial recognition, voice recognition	Mobile banking app; ATM transactions
**HSBC Hong Kong**	Fingerprint, facial recognition	Mobile banking app
**RBC (Royal Bank of Canada)**	Fingerprint, facial recognition	Mobile banking app
**NAB (National Australia Bank)**	Fingerprint, facial recognition	Mobile banking app

From Table 9 we note that: Fingerprint and Facial recognition are the two most used biometric mechanisms probably because most of the smartphones now support them and they are highly accepted by users. Voice recognition is rarer, and more often in mobile-based banking. Palm veins are quite unusual, but there is higher security and usage in physical (store for payments) authentication scenarios. Mobile banking for convenient and secure login is the most common area of biometric applications. Some banks (for example, DBS Bank) merge several biometric devices, for example: fingerprint, face and voice – suggestive of a multi-modal approach in security improvement.

**Figure 7.**
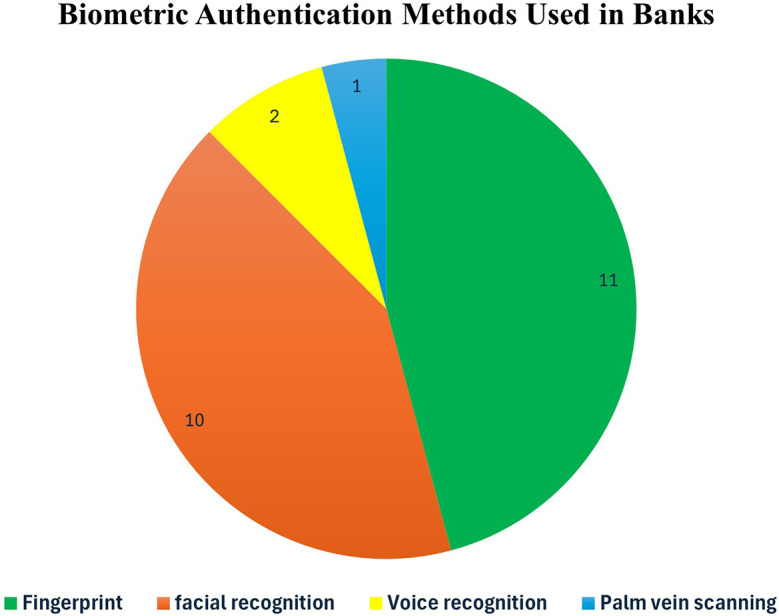
Distribution of biometric authentication technologies (e.g., fingerprint, facial, voice, palm) adopted by major banks in the world, reflecting adoption trends and popularity in mobile banking services.

Figure 7 depicts visual representation of how often each of the biometric authentication methods are used by the banks. As you can observe, fingerprints and facial recognition outrun the competitors, while voice and palm vein scanning are far rarer.

### 4.4 Usability and user perception challenges and limitations

The literature shows that there is a stark tradeoff between strength of security and usability. As biometrics increase protection, the ineffective user experience will decrease adoption and efficacy. The banks must focus on usability testing, offer alternative, safe options (i.e., token generated by the device), and increase transparency regarding the use of biometric data.

The usability and how often it is perceived by the user are important issues that determine the significance and adoption of biometric authentication mobile banking techniques. As indicated in various other studies ^156, 157^ one of the issues many users face when using biometric systems is irritation related to the unreliability of these systems, such as inability to recognize their fingerprint when their fingers are wet, in situations of skin damage, or due to technical purposes of sensors. In the same light, facial recognition has been observed to perform poorly in low-light situations or when the user is wearing glasses or face mask. Such technical shortcomings may undermine the trust in the system and make people turn off the security features or switch to the less safe options (e.g., back to the passwords or PINs).

On top of technical problems, user perception plays an important part in the adoption of biometrics. A study conducted by ^158^ has revealed that a high percentage of users were worried about the personal safety and confidentiality of biometric data particularly when this data was stored on cloud-based platform instead of being secured on the gadget. The level of confidence in banks and their confidentiality of information was a deciding factor as to whether the user activated biometric authentication. Moreover, some studies like, ^38^ have pointed out the issue of the inaccessibility of MFA especially to ageing people and individuals with disabilities who might have a hard time using or comprehending some MFA techniques. Overall, literature points at the necessity to design authentication systems in a way that composes security and simplicity to use, availability, and visibility, so that security apparatus will not be an obstacle to user interaction. Online banking user authentication techniques and cyber threats maintain an uncertain outlook, but the resulting risks remain massive. ^58^

In this section we answered **RQ4** which stated: “What is the key **usability, privacy, and user acceptance challenges** related to biometric authentication in mobile banking?”.

These challenges represent the main difficulties that must be addressed when enhancing the security of online banking systems, in addition to user authentication procedures. We have classified the most important challenges as follows:

**4.4.1 Artificial intelligence challenge**

The adoption of artificial intelligence through machine learning by cyberattacks presents a threat to online banking infrastructure, which includes network breaches and defeats detection systems. The implementation of ML involves several methods for breaching online banking authentication systems. ^159– 161^

**4.4.1.1 Bias in biometric systems**

Biometric systems powered by artificial intelligence produce irregular results when processing users from diverse population demographics, including ethnicities, as well as gender and age groups. A lack of diversity in training data can result in unfair FAR or FRR errors that produce trust and legal challenges among specific users. ^162^

**4.4.1.2 Adversarial attacks**

Information security risks exist because AI authentication systems can be deceived through adversarial examples that adversaries specifically generate to trick them (such as manipulated fingerprints, deepfakes or altered patterns). Researchers aim to build authentication models with resistance to manipulation because this remains a critical research topic. ^125, 163, 164^

**4.4.1.3 Deep fakes can be produced**

Artificial deep fakes demand ML techniques for their production. Artificial works labeled deep fakes combine someone else’s likeness by replacing it with extant images and footage. Any recorded online banking registration activity can be faked through deep fakes to steal user login credentials used in the fake video. ^163, 165– 167^

**4.4.1.4 ML technology**

ML enables the automation of assaults against banking systems that operate through the internet. The automation of attacks targeting online banking systems becomes possible through the application of ML techniques for addressing guessing attacks as well as brute-force attacks and DoS attacks. ^144^ The attackers benefit from simpler ways to run successful assaults against online banking infrastructure. Phishing attacks can be launched with the assistance of ML. ^168^ Phishing attacks use deceptive methods to force users to reveal their critical account information, including bank card numbers and network access secrets. AI facilitates the development of convincing phishing emails along with corresponding websites that target users. ^159^ These attacks become more deceptive because they offer a higher probability of deceiving users.

ML provides banks with tools to protect their systems from cyberattacks, enabling them to enhance the security and resilience of their systems. By utilizing ML, banks enhance both their risk management and compliance practices to detect and prevent fraud together with malware and phishing attacks. ^169^ The application approach of ML determines whether it offers either risk or an opportunity to use online banking systems. ^170^

**4.4.1.5 High computational and energy demands**

The functionality of real-time authentication based on AI processing through onboard device resources is restricted to older and less expensive mobile devices. Cloud storage introduces new security risks during processing combined with greater processing delays. ^171^

**4.4.1.6 Explainability and transparency**

The issue of explainability and transparency in mobile banking deserves attention because biometric authentication systems are often complicated. Since these systems heavily depend on complicated algorithms, the AI-based and machine learning algorithms to make authentication decisions, unsuspecting individuals and even the systems operators are often locked out of the process of how such decisions are made. Failure to find an obvious justification in denying access or scoring suspicious users may cause frustration, mistrust, and losses to user abandonment. In addition, regulatory and ethical aspects, audit, accountability, and determination of fairness are also critical regarding transparent systems, especially when it is biometric data of a sensitive nature. Improving explainability will enable developers to pinpoint and redress errors or bias and give users a more effective understanding of how their data are utilized, enhancing trust and confidence in the mobile banking authentication in general. ^171, 172^

**4.4.1.7 Spoofing and presentation attacks**

The presented attack and spoofing vulnerability are one of the greatest disadvantages of biometric authentication in mobile banking systems. These attacks consist of hoodwinking the system by fueling fake or imaginary biometrics, including silicon fingerprints, ^132^ photographs with a high resolution of the face, or even complex masks made of 3D printers. ^173^ As an example, can an attacker lift a fingerprint off a surface and re-mold it in gelatin or latex material or defeat facial recognition by having a video replay or a 3D mask. ^174^ Most bio-metric systems have attempted to counteract these threats by utilizing liveness except tools, which might include blinking or feel of the skin texture, although not universally and reliably as in the case with all devices. Spoofing attacks can succeed better in low end smartphones or applications with weak security. Also, the use of liveness detection may affect user experience in some cases, which makes developers turn it off or modify it in some way. ^43^ With ever evolving spoofing techniques such as usage of deep fakes and masks embedded with motion, the present-day biometric systems are struggling to provide secure authentication. This does not only endanger user accounts to unauthorized access but also jeopardizes the user faith; it raises legal and regulatory issues to financial institutions. Implementing multimodal biometric authentication, constant surveillance and AI-based spoof protection is essential in building solid mobile banking security. ^175^

**4.4.1.8 Dependency and hardware limitations**

In mobile banking, the performance of biometrics depends highly on the hardware capacity of their devices. Premium smartphones are additionally obvious to have improved sensors, such as depth cameras or ultrasonic fingerprint readers, ^176^ which enable more authentication with high degrees of accuracy and safety. On the other hand, the cameras that come with the low-end or older devices are of basic 2D type or capacitive sensors, and these are more error prone and could be more easily spoofed. Such hardware mismatches may cause uneven authentication experiences, false acceptance rate/false rejection rate and exclusion of users with an outdated device. ^177^ Also, the scarcity of hardware support makes the process of developing apps harder and does not allow implementing higher levels of security, which, in turn, makes mobile banking platforms less secure and readily accessible.

**4.4.2 False Acceptance/False Rejection Rates (FAR/FRR)**

**Figure 8.**
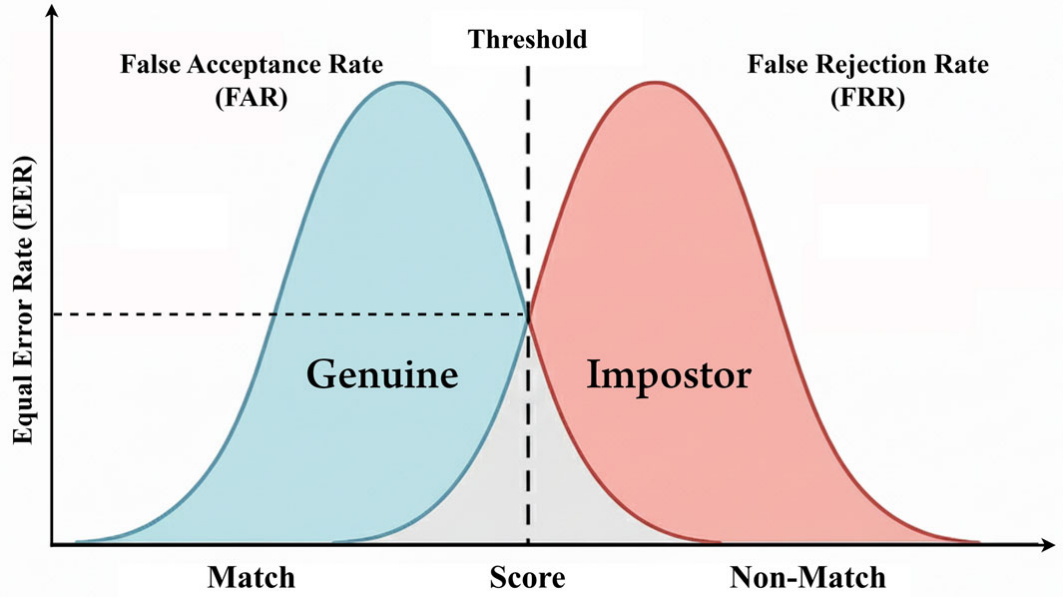
Illustration of the trade-off between FAR and FRR in biometric authentication.

FAR and FRR are some of the errors ( Figure 8), which make biometric authentication systems vulnerable. FAR is defined as access given to unauthorized users when it is not supposed to be which is wrong and FRR is defined as being denied by the unauthorized user when it is not supposed to be the case. Such errors may be caused, among others, with improper lighting, inclusions or damage of sensors, alterations of physical appearance of the user (age, injury, facial hair), or inconsistent behavior of the sensors among different devices. When FAR is high this compromises security whereas when FRR is high this affects usability and user satisfaction, two important functions in mobile bank applications. Designing a workable balance between these rates is a major difficulty in the design of biometric systems. ^177^

**4.4.3 Balancing security and usability**

This right balance between security and usability is one of the major issues of implementing biometric authentication methods giving in mobile banking. Although greater security can be brought about by higher quality security procedures, e.g., through multiple authentication factors or high-quality liveness detection, this can also serve to deliver friction, delay or confusion to users. Conversely, simplified forms of authentication can provide a better experience but can leave systems at a higher risk of attack. To give an example, high tires of login using multiple steps can be strenuous to users and decrease the usage of the application and by contrast, using biometrics as the only mode of verification can isolate users with certain accessibility challenges. To achieve effective authentication, one therefore must balance a range between sound protection over threats and not jeopardizing ease of use and accessibility by the users. To do that, it will be necessary to have adaptive authentication mechanisms, user-centered design, and continuous assessment of security point risks as well as user behavior. ^178, 179^

**4.4.4 Regulatory and ethical compliance**

Regulatory and ethical compliance plays an important role when implementing biometrics authentications in mobile banking. The legal environment is very complicated, and financial institutions have to comply with international data protection regulations including the **General Data Protection Regulation (GDPR),** Regulation (EU) 2016/679 of the European Parliament and of the council on data protection and privacy and the California Consumer Privacy Act of 2018 (CCPA), enacted by the California Legislature to enhance consumer data privacy protections in the United States. ^161^ These laws obligate organizations to obtain informed consent from users, reduce the amount of data collected, store information in a safe manner and make the processing of the biometrics open. Banks also have an ethical obligation to stop misuses or discrimination through systems that provide poor results according to a demographic group. A failure to fulfill these responsibilities may lead to legal action and damage to reputation, an economic impact and loss of the trust of the users. To adhere to it, organizations are required to incorporate privacy-by-design, conduct regular system audit to determine its fairness and non-discriminatory nature, and ensure that it grants users full control over their personal biometric information. ^147^

**4.4.5 User privacy challenge**

Online banking authentication through the BBA, including fingerprint authentication, presents the highest security level because attempts to forge or steal biometric data prove very difficult. The application of BBA includes privacy-related problems. A biometric database breach of a bank will give hackers the ability to obtain all the authenticity data of its banking clients. Customers have reservations about banking establishments and other organizations storing their biometric information. Banks that utilize BBA require proper protection of biometric data that belongs to their clients. Banks must secure data against unlawful handling through preventative measures. A privacy policy needs to exist that explains to clients how their biometric information will be handled. ^126^

**4.4.6 System compatibility challenge**

Financial institutions must transform their current technology platforms and authorize procedures to welcome different authentication approaches, such as biometrics along with tokens and MFA, for online banking access. Additionally, merchants should approve of their payment vendors, who assist in online authentication. ^179^

**4.4.7 System usability challenge**

The user authentication sector faces a conventional challenge because securing systems becomes riskier when making authentication methods easier to use. The implementation of layering represents a security improvement approach for user authentication systems. ^98^ MFA requires multiple security checks that combine multiple factors such as biometric authentication, passwords/PIN, OTP and/or other factors. ^73^ Layering is founded on the fact that if one safeguard is breached, other levels of security will still protect the system from the undesired users. The number of layers determines better system protection but creates a declining usability experience. The authentication procedure becomes more challenging with each additional layer, which ultimately leads to user frustration. ^178^ Our system enhances security when we use multiple authentication layers, although usability decreases at the same time. Security and usability must be balanced when choosing authentication methods according to the required security measures.

## 5. Limitation

This systematic review has several limitations. First, a formal risk-of-bias or certainty assessment was not conducted because the included studies are highly heterogeneous in terms of datasets, evaluation protocols, biometric devices and performance metrics. Such variations make the use of standardized tools (e.g., RoB, GRADE) unsuitable for technical and engineering research. Second, a meta-analysis was not possible because of the lack of comparable quantitative data across studies, because the reporting formats and evaluation measures (e.g., EER, FAR, FRR) differ significantly. Third, only studies published in English and available through selected digital libraries were included, which may introduce publication or language bias. Despite these limitations, the current biometric-based authentication research is comprehensively synthesized in this review, and a structured analysis is presented to guide future directions in mobile banking security.

## 6. Conclusion and future directions

This study analyzes biometric authentication methods implemented in mobile banking from 2020–2025 via an SLR. The review examined five essential topics, including mobile banking authentication methods and security risks, the biometric authentication methods deployed by major banks worldwide, usability challenges and limitations, and future directions.

The authentication methods for online mobile banking users can be categorized into four categories: BBA, KBA, PBA and MFA methods. Each part contains a variety of authentication methods with varying weaknesses, strengths, and implementation. KBA methods depend on user knowledge, such as PIN, passwords and security questions, to verify their identity. The verification process under KBA authentication methods depends on the information that the user has, which includes passwords, PINs and security questions. BBA methods identify users through specific measurements of biological markers known as physiological traits, which include fingerprints, facial identification, iris scanning, hand geometry, vein and behavioral biometrics, including voice recognition, keystroke, EEG, signatures, hand movement patterns and continuous authentication, such as touch screens. Users access their accounts through PBA methods by relying on authentication tokens that include OTP authentication, which sends exclusive temporary codes to users through SMS, e-mail or other applications and covers security keys/USB and wearable devices. The authentication methods employ different procedures that do not belong to either the KBA or the BBA or PBA categories. Among these authentication examples are MAC addresses, IP addresses, proxies, QR codes, geolocation data and CAPTCHAs. MFA frameworks enhance security by implementing multiple authentication approaches that belong to different categories. When banks implement multiple authentication methods, they build up their system security while developing a multilayered protective system, which makes it difficult for attackers to break in. The multiple layers ensure system protection because each layer provides additional security if an attacker manages to breach one defense mechanism.

This research analysis examined multiple types of cyber threats that target online banking systems while discussing methods to bypass authentication protection measures. Multiple online banking threats include malware attacks, social engineering techniques and phishing attacks, MitM attacks, DoS attacks, session hijacking, weak passwords, keyloggers, SQL injections and replay attacks. The research examined different authentication methods used by well-known banks to determine secure practices for protecting online banking accounts.

By discussing the advantages and disadvantages of the user verification methods used in online payments, this research analyzes the biometrics authentication methods that are implemented in mobile banking; thus, the security of mobile banking has increased because biometrics verify users by checking their unique characteristics, including fingerprints and facial recognition. The use of biometric enhances mobile banking security with superior identity verification, which results in higher access speed in addition to better fraud protection. The security and user authentication process of online banking systems need improvement through the consideration of four key challenges: artificial intelligence, FAR/FRR, balancing security and usability, regulatory and ethical compliance, user privacy challenge, system compatibility challenge and system usability challenge. This research has several major limitations because it relies on literature reviews about online mobile bank account authentication.

The following section answered **RQ5** which stated: “What are the **limitations and future research directions** in improving biometric-based authentication for secure and convenient mobile banking?”. These limitations are doesn’t use fusion of biometrics in mobile banking, digital wallets, blockchain and wearable devices such as rings. Future research should include blockchain authentication, fusion techniques, e-wallets authentication and wearable devices, and other future directions related to security and privacy, such as the following:

1- Adaptive and Context-Aware Authentication: Research should evaluate methods for authentication systems to change security levels through real-time threat analysis and behavioral information and environmental signals (such as location status and device health status).
2- Privacy-Preserving Biometric Systems: Biometric data protection research must focus on security strategies that start from data collection through storage and transmission phases since privacy-protecting techniques such as homomorphic encryption and secure multiparty computation enter the market.
3- Threat Modeling in Evolving Environments: The ongoing analysis of threats should predict potential weaknesses that arise from new technology implementations such as embedded banking combined with AI fraud detection systems and wearable biometric scanning.
4- Future Use of Multimodal Fusion: Further studies of highly integrated fusion techniques capable of integrating various biometric identifiers (e.g., fingerprint and face) with the context in a way that maximizes security and usability must be investigated. An AI-driven decision model is likely to enable more reliable and user-friendly authentication in high-risk contexts such as mobile banking and e-government services in the future through the fusion of AI with various modalities whose weights are adaptively changed by their reliability in real-time.

The research results will appeal to multiple groups, such as financial organizations and official government agencies, together with academic researchers. The resulting insights will enable researchers to create security systems as well as educational content that safeguards users from performing online banking activities.

## Data Availability

All data supporting the findings of this systematic review have been deposited in the Zenodo public repository and are openly accessible under the CC0 1.0 Universal license. The dataset includes:
• The completed **PRISMA 2020 checklist**
• The **PRISMA flow diagram**
• The **SLR dataset** containing all 97 included studies with extracted variables **Repository:** Zenodo **Title**: *PRISMA Checklist, Flow Diagram, and SLR Dataset for “A Systematic Literature Review on Biometric Authentication in Mobile Banking”* **DOI**: https://doi.org/10.5281/zenodo.17744117 ^180^ **License:** CC0 1.0 Universal These materials provide full transparency and allow complete reproducibility of the review process.
