# Supplementary material for: A Systematic Literature Review on Biometric Authentication in Mobile Banking
Source: F1000Res. 2026 May 7;15:5. Originally published 2026 Jan 6. [Version 2] doi: 10.12688/f1000research.173855.2 (PMC13237553; doi:10.12688/f1000research.173855.2)
Supplement: Peer review history

# Reviewer report: Reviewer response for version 2

**DOI:** 10.5256/f1000research.199045.r482373

**Published:** 2026-06-04

**Authors:** Wojciech Wodo

**Affiliations:** 1 Wroclaw University of Science and Technology, Wroclaw, Poland

My previous review indicated that the manuscript required substantial improvement. The authors have introduced minor revisions, which address my comments only partially. Their responses suggest a disagreement with some aspects of my critique, leading them to refrain from implementing corresponding changes in the paper. In fairness this would have required a substantial rewrite. 

 I maintain that my original comments remain valid. However, I have also taken into consideration the more favorable evaluation provided by the second reviewer. In light of this, I have decided to agree that the paper can be indexed - even though it makes less of a contribution than what I would have liked.

Are the rationale for, and objectives of, the Systematic Review clearly stated?

Partly

Is the statistical analysis and its interpretation appropriate?

No

If this is a Living Systematic Review, is the ‘living’ method appropriate and is the search schedule clearly defined and justified? (‘Living Systematic Review’ or a variation of this term should be included in the title.)

No

Are sufficient details of the methods and analysis provided to allow replication by others?

Yes

Are the conclusions drawn adequately supported by the results presented in the review?

Partly

Reviewer Expertise:

cybersecurity, biometrics, digital identity, electronic & mobile banking security

I confirm that I have read this submission and believe that I have an appropriate level of expertise to confirm that it is of an acceptable scientific standard.

# Reviewer report: Reviewer response for version 2

**DOI:** 10.5256/f1000research.199045.r482374

**Published:** 2026-05-25

**Authors:** Geeta Sandeep Nadella

**Affiliations:** 1 University of the Cumberlands, Williamsburg, USA

Thank you for the opportunity to review the revised version of your manuscript. Having carefully examined your response to my previous comments and the corresponding changes made to the manuscript, I am pleased to confirm that you have addressed all of my constructive concerns in a thorough and satisfactory manner. The strengthened synthesized discussion of quantitative findings now offers much clearer practical insights, particularly regarding the specific conditions under which different biometric modalities perform well or exhibit limitations. Your explicit linking of usability and privacy challenges to the real‑world implementation strategies of major banks, including the use of layered controls such as OTP, device recognition, and step‑up authentication, successfully bridges the gap between academic analysis and industrial practice. Furthermore, your forward‑looking recommendation for more standardized evaluation protocols in future primary research is a valuable contribution that will help the field move toward more comparable and reproducible evidence. The manuscript is now more coherent, analytically deeper, and practically relevant, while retaining its clear methodological rigor and comprehensive scope. I believe it makes a timely and useful contribution to the literature on biometric authentication in mobile banking. I have no further reservations and recommend that the manuscript be accepted without additional revisions. Congratulations on your thorough and responsive work.

Are the rationale for, and objectives of, the Systematic Review clearly stated?

Yes

Is the statistical analysis and its interpretation appropriate?

Yes

If this is a Living Systematic Review, is the ‘living’ method appropriate and is the search schedule clearly defined and justified? (‘Living Systematic Review’ or a variation of this term should be included in the title.)

Not applicable

Are sufficient details of the methods and analysis provided to allow replication by others?

Yes

Are the conclusions drawn adequately supported by the results presented in the review?

Yes

Reviewer Expertise:

MIS, Artificial Intelligence, HCI, Cybersecurity, Business and IT alignment

I confirm that I have read this submission and believe that I have an appropriate level of expertise to confirm that it is of an acceptable scientific standard.

# Reviewer report: Reviewer response for version 1

**DOI:** 10.5256/f1000research.191708.r450385

**Published:** 2026-02-14

**Authors:** Wojciech Wodo

**Affiliations:** 1 Wroclaw University of Science and Technology, Wroclaw, Poland

In my opinion, preparing the review paper is quite a challenging task, usually much more demanding than a regular research article, as it requires an extensive knowledge of the domain, a deep understanding of details, nuances, and a broad perspective of the described area. The contribution of the review paper is sometimes hard to grasp; it requires an in-depth analysis of the state-of-the-art literature and often internal (not publicly available) industry or business practices and the rationale behind them. To provide a vital contribution to the public, the author of the review paper should demonstrate a comprehensive understanding not only of the technology used but also of regulatory frameworks, compliance issues, as well as industrial or business-specific conditions.

**SOME BASIC INFORMATION ABOUT THE PAPER**

 According to the authors **the scope of the paper** is:

 This systematic literature review based on PRISMA methodology, which covers studies from 2020 to 2025, provides a critical review of biometric authentication methods used in mobile banking.

**Reviewer’s remark:**

 Regarding methodology, authors decided to search four databases: ScienceDirect, Scopus, IEEE, and Google Scholar in order to select papers for analysis. The whole process of selection and eligibility criteria as well as exclusion/inclusion approach was clearly presented. However, in my perspective this extremely important step is flawed by design, because authors limited themselves from the very beginning in learning insights from the industry/business perspective by excluding white papers, technical reports/notes, or from in-depth analysis presented in theses or book chapters. I cannot find any justification for such an approach in the paper and I find it restrictive to the study.

**Objectives are:**

 1. An analysis of the existing approaches, security risks and implementation practices adopted by major banks across the world.

 2. A detailed overview of the current advances, key issues, and emerging research directions, which will give valuable insight to the development of secure and easy-to-use authentication systems in mobile banking.

**Reviewer’s remark:**

 Motivation is somewhat not clear to me, authors claimed that they did not find a systematic literature review that focused specifically on the uses of BBA in mobile banking. However, the mere fact that no study exists should not be an end in itself for preparing one. The authors did not clearly state what motivated them to take up this topic and what their main idea and goal for the study was. All information is very generic and superficial in this regard. We can find information that they wanted to analyze biometric authentication methods, but we do not know why.

**Intended audience:** The present manuscript is aimed at a wide audience of both academic researchers and industry practitioners.

**Reviewer’s remark:**

 I cannot agree with authors in this regard, after my evaluation I would say that paper targets students or entry-level professionals and serve as an educational material, or introductionary material to get oriented in fundamentals of described domain.

 Authors stated **five main research quesitons**:

 1. Which biometric authentication methods are currently used in mobile banking systems?

 2. What are the main security threats and vulnerabilities affecting biometric authentication in mobile banking?

 3. How do major banks worldwide implement and integrate biometric authentication into their mobile banking applications?

 4. What is the key usability, privacy, and user acceptance challenges related to biometric authentication in mobile banking?

 5. What are the limitations and future research directions in improving biometric-based authentication for secure and convenient mobile banking?

**REVIEWER’S EVALUATION**

 The paper tackles an undeniably important and interesting topic, and the authors demonstrate a transparent methodology of work, selecting multiple papers for analysis. The structure of the review is easy to follow, and there are formulated research questions and appropriate sections. The attached exhibits and tables are prepared in a neat manner. I have no objections to the formal or editorial aspects of the work; however, I have some substantial reservations about its essential content and the way of presenting the results and insights of the study.

**Major remarks:**

 1. One cannot elaborate on financial or payment market as a homogenous one, as there are different regulatory frameworks on risk, payments, identity management, AI or personal data protection that should be considered when applying technological solutions. These issues tremendously impact the bank and payment industry. Discussion on security mechanisms used in banking should consider wider context of regulatory domain, market maturity and users’ experience.

 For instance, according to PSD2 and the accompanying RTS, introducing SCA (Strong Customer Authentication) for a wide range of financial transactions is mandatory since the directive came into force in 2019 in the European Union market. The scope of the regulation tackles not only strengthening the authentication by enforcing 2FA mechanisms but also OTP binding with transaction data and time validity, so that we can achieve the so-called "dynamic linking" property and expiration time of the token.

 2. Presentation Attacks and Injection Attacks are very powerful threats for biometric-based systems these days, and with the aid of AI, they are even more dangerous, as it is challenging to differentiate the genuine identity from the impostor. The authors claimed for instance that:  “Biometrics offer very high resistance against replication and remain difficult to counterfeit” (page 7) which is clearly incorrect. Almost every vendor of eKYC solutions for the high-risk industry, like banking or gambling, faces this issue.

 NIST is running a project on Face Analysis Technology Evaluation (FATE), which one of the area is PAD (Presentation Attack Detection) - https://pages.nist.gov/frvt/html/frvt_pad.html, where are competing the best of the best players in the market and still are not good enough in detecting the frauds.

 3. The paper contains a multitude of basic definitions and explanations of concepts that are obvious in the world of computer security. The paragraphs devoted to individual cybersecurity risks are very short and superficial.

 4. The criteria for selecting the banks included in the study are unknown, and relying solely on information obtained from a literature review may, in this case, result in an incomplete or inaccurate picture of the security solutions and challenges in individual banks. In my opinion, it would be necessary to verify/ensure that the information presented reflects the actual state of affairs.

**Minor remarks:**

 1. Regarding Introduction section and BBA bullet point, it would be vital to mention about "cancelable biometrics" approaches there

 2. Information provided by Table 3 is too short to be useful, reader without reading the cited paper cannot grasp the essence of the solutions.

 3. Figure 5 or Table 4 are fancy, but not meaningful, there is no vital data provided through them

 4. Table 5 requires some editorial work – e.g. wrong spacing

 5. In Table 6 a lot of data is neither available nor applicable

 6. What is the aim of presenting data in Table 8 and Table 9?

**CONCLUSION OF THE REVIEW**

 The study fails to provide high-level insights on the presented subject; there is no vital contribution from the authors in building the wider context for the examined area and in drawing out meaningful takeaways.

 The presented material is rather a well-done scrutiny job than in-depth analysis and synthesis of general and more abstract results. The reader can find the answers to the questions What? and sometimes How? but there is no Why? and So what? questions addressed.

 Almost all of the included exhibits are a reflection of the information provided in the text, without any additional value to the reader - more like for an aesthetic effect.

 Despite an undeniable load of work done by the authors, the submitted manuscript can be treated rather as preliminary work than ready to go, and in my view, it is not suitable for publication in such a form. It requires additional layers of processing gathered data and formulating conclusions and generalizations.

 Source selection should also be extended by previously excluded types of bibliographic items, such as industry/business whitepapers, technical reports/notes, or theses and book chapters, as well as or legal acts or international standards containing extremely important data and insights and providing necessary context for the elaborated area.

 My general impression is that paper lacks its leading specific angle of looking at the area of interest. Analyzing the material through a “specific and more in-depth perspective” would allow for interesting conclusions and observations that would be more concrete and specific and more interesting to the audience.

Are the rationale for, and objectives of, the Systematic Review clearly stated?

Partly

Is the statistical analysis and its interpretation appropriate?

No

If this is a Living Systematic Review, is the ‘living’ method appropriate and is the search schedule clearly defined and justified? (‘Living Systematic Review’ or a variation of this term should be included in the title.)

No

Are sufficient details of the methods and analysis provided to allow replication by others?

Yes

Are the conclusions drawn adequately supported by the results presented in the review?

Partly

Reviewer Expertise:

cybersecurity, biometrics, digital identity, electronic & mobile banking security

I confirm that I have read this submission and believe that I have an appropriate level of expertise to state that I do not consider it to be of an acceptable scientific standard, for reasons outlined above.

# Response

**Published:** 2026-04-18

**Authors:** Hasan Naji

**Affiliations:** Computer Science, University of Mosul College of Computer Sciences and Mathematics, Mosul, Nineveh Governorate, Iraq

**Response to Reviewer**

 We sincerely thank the reviewer for the careful reading of our manuscript and for the detailed, thoughtful, and constructive comments. We also apologize for the delay in submitting our revised response, which was due to a temporary health issue. We greatly appreciate your patience and understanding.

 We are especially grateful for the reviewer’s emphasis on the need for stronger contextualization, deeper synthesis, and clearer articulation of the practical significance of the review. We have carefully considered these comments and revised the manuscript accordingly, while also acknowledging certain scope limitations of the present study. Our point-by-point responses are provided below.

**Major Comment 1:**

*The paper does not sufficiently consider the wider regulatory, compliance, and market context; banking and payment markets are not homogeneous, and frameworks such as PSD2/SCA should be considered.*

**Response:**

 Thank you for this important observation. We agree that regulatory and compliance frameworks play a major role in shaping authentication practices in banking and payment systems, and that the financial sector cannot be treated as fully homogeneous across jurisdictions. In the revised manuscript, we strengthened the discussion of real-world implementation by more explicitly linking authentication practices to practical banking deployment conditions and by expanding the discussion of security–usability trade-offs in operational settings. We also acknowledge that a deeper comparative treatment of jurisdiction-specific regulatory frameworks, such as PSD2/SCA and related compliance requirements, would further strengthen the broader contextual framing. We therefore recognize this as an important direction for future extension of the review and as a limitation of the current study’s scope.

**Major Comment 2:**

*The manuscript underestimates the seriousness of presentation and injection attacks, particularly in the AI era, and some statements about biometric resistance to counterfeiting are too strong.*

**Response:**

 We thank the reviewer for highlighting this point. We agree that presentation attacks and related spoofing threats are among the most critical challenges facing biometric systems in contemporary high-risk sectors such as banking. In response, we revised the manuscript to place greater emphasis on spoofing resistance, liveness detection, and the limitations of reported biometric performance. In particular, the revised quantitative discussion now clarifies that performance results are often difficult to generalize across real-world deployment conditions and that only a limited number of reviewed studies explicitly evaluated presentation attack detection or spoofing resistance. We also carefully reconsidered overly broad wording regarding biometric resistance to replication and recognize the reviewer’s point that such statements must be qualified in light of current attack capabilities.

**Major Comment 3:**

*The paper contains too many basic definitions and some cybersecurity risk discussions are short and superficial.*

**Response:**

 Thank you for this observation. We understand the reviewer’s concern and acknowledge that the manuscript includes foundational explanatory material intended to make the review accessible to a broad readership. At the same time, we appreciate that for expert readers, some of these sections may appear introductory. In the revised version, we sought to improve the analytical depth of the manuscript by strengthening the synthesis sections, especially in relation to quantitative findings, implementation practices, and practical implications. We also recognize that further tightening of basic explanatory material and additional expansion of higher-level synthesis could improve the manuscript further, and we appreciate this guidance.

**Major Comment 4:**

*The criteria for selecting the banks are unclear, and relying only on literature sources may not fully reflect actual banking practices.*

**Response:**

 We appreciate this important point. The bank-related analysis in the manuscript was intended as an illustrative synthesis of practices reported in the reviewed and cited sources, rather than as a definitive audit of the currently deployed security architecture of each institution. We agree that real-world banking implementation may evolve rapidly and may not always be fully captured in academic literature alone. In the revised manuscript, we improved the framing of this section to better reflect its interpretive purpose and to avoid overstating the completeness of the bank comparison. We also acknowledge that future work would benefit from expanding the evidence base to include additional industry and regulatory sources where appropriate.

**Major Comment 5:**

*The rationale and motivation are not sufficiently clear, and the paper does not adequately answer the questions Why? and So what?*

**Response:**

 Thank you for this valuable comment. We took this concern seriously. In response, we strengthened the manuscript’s framing by clarifying that the purpose of the review is not merely to catalogue biometric methods, but to synthesize how biometric authentication is being used in mobile banking, what practical limitations remain, where current evidence is methodologically uneven, and which approaches appear most relevant for secure and usable deployment. We also strengthened the conclusion so that the review more clearly communicates the practical significance of the findings, particularly the observation that real-world banking practice increasingly favors layered and adaptive authentication rather than reliance on a single biometric factor.

**Major Comment 6:**

*Source selection should be expanded to include white papers, technical reports, theses, book chapters, legal acts, standards, and related materials.*

**Response:**

 We appreciate this suggestion and agree that such sources can provide valuable insight, especially for industry practices, regulatory interpretation, and deployment realities. However, in the present review, we intentionally limited the formal evidence base to English-language journal and conference publications in order to maintain a more consistent and transparent selection framework under the chosen systematic review methodology. We acknowledge that this decision narrows the scope of the review and may exclude valuable non-traditional or industry-oriented sources. We have therefore treated this issue more explicitly as a limitation of the present study and agree that future reviews could usefully adopt a broader evidence strategy incorporating regulatory, industrial, and technical documentation.

**Minor Comment 1:**

*Cancelable biometrics should be mentioned in the Introduction.*

**Response:**

 Thank you for this helpful suggestion. We agree that cancelable biometrics are relevant in the broader privacy and security discussion of biometric systems, particularly because they address the problem of revocability when biometric templates are compromised. We appreciate the value of this point and recognize it as an important concept for strengthening the introductory framing.

**Minor Comment 2:**

*Table 3 is too brief to be useful.*

**Response:**

 We appreciate this comment. Table 3 was designed to provide a concise overview of the reviewed studies and their core focus areas rather than a full technical exposition of each method. However, we understand the reviewer’s concern that brevity may reduce interpretive value for readers. In the revised manuscript, we sought to strengthen the surrounding synthesis so that the table functions more clearly as a reference aid within a broader analytical discussion.

**Minor Comment 3:**

*Figure 5 and Table 4 are visually appealing but not very meaningful.*

**Response:**

 Thank you for this observation. The intention of Figure 5 and Table 4 was to organize and visualize the diversity of authentication combinations reported across the literature, especially the interplay between biometric, knowledge-based, possession-based, and hybrid approaches. We acknowledge, however, that their analytical value depends on clear integration with the discussion. In the revised manuscript, we worked to improve that integration and appreciate the reviewer’s comment regarding the importance of ensuring that visual elements contribute interpretive value beyond presentation.

**Minor Comment 4:**

*Table 5 requires editorial refinement.*

**Response:**

 Thank you. We appreciate this observation and carefully reviewed the manuscript for editorial consistency, including tabular formatting.

**Minor Comment 5:**

*Much of the data in Table 6 is unavailable or not applicable.*

**Response:**

 We agree that this is an important limitation. Table 6 was included precisely to make visible the inconsistency and incompleteness of reporting across the reviewed literature. In the revised text, we more explicitly state that incomplete reporting of FAR, FRR, EER, liveness evaluation, and spoofing resistance is itself an important finding of the review and one of the reasons why stronger standardization is needed in future primary studies.

**Minor Comment 6:**

*The purpose of Table 8 and Table 9 is unclear.*

**Response:**

 Thank you for raising this point. The purpose of these tables was to connect the academic literature with practical banking deployment patterns and to illustrate which biometric approaches appear most visible in major banking contexts. In the revised manuscript, we clarified this linkage more explicitly, particularly in the discussion of how banks balance usability and security by embedding biometrics within broader MFA strategies rather than using them in isolation.

 We once again thank the reviewer for the detailed and thought-provoking feedback. The comments helped us identify important areas where the manuscript required stronger synthesis, clearer framing, and more careful qualification of its claims. We believe that the revisions have significantly improved the clarity, depth, and practical relevance of the review.

# Reviewer report: Reviewer response for version 1

**DOI:** 10.5256/f1000research.191708.r450383

**Published:** 2026-01-29

**Authors:** Geeta Sandeep Nadella

**Affiliations:** 1 University of the Cumberlands, Williamsburg, USA

The review is well-structured, methodologically sound, and clearly reports its rationale, methods, and conclusions. It does not involve statistical analysis beyond reporting existing metrics, and it is not a living review. This systematic literature review provides a valuable and timely synthesis of research on biometric authentication in mobile banking from 2020 to 2025. The rationale and objectives are clearly articulated, establishing a strong foundation for the work. The methodology is rigorously described using the PRISMA framework, with transparent reporting on databases, search strings, and inclusion criteria, which makes the process highly replicable. The analysis successfully addresses the stated research questions, offering a comprehensive overview of methods, threats, and banking practices, and the conclusions are well-supported by the presented evidence.

 To further strengthen this already solid work, several constructive avenues are worth considering. While the review effectively categorizes biometric methods and their performance metrics (e.g., FAR, FRR, EER), a more synthesized critical discussion of these quantitative results would be beneficial. For instance, highlighting the specific conditions under which certain biometrics excel or fail, based on the aggregated data, could offer more actionable insights for practitioners. Additionally, the section on usability and privacy challenges is comprehensive, but it could be enhanced by more explicitly linking these challenges to the specific implementation strategies of the banks analyzed earlier. Discussing how leading banks are practically navigating the trade-off between security strength and user experience would bridge the analysis and its real-world application more powerfully.

 Finally, the limitation regarding the heterogeneity of studies is well-noted. A forward-looking recommendation could be to explicitly call for more standardized evaluation protocols in future primary research to facilitate more robust comparative synthesis in subsequent reviews. Overall, this is a commendable and useful piece of scholarship that makes a clear contribution to the field by organizing a rapidly evolving body of knowledge. With some refinement in synthesizing quantitative findings and connecting analysis threads, its impact could be even greater.

Are the rationale for, and objectives of, the Systematic Review clearly stated?

Yes

Is the statistical analysis and its interpretation appropriate?

Yes

If this is a Living Systematic Review, is the ‘living’ method appropriate and is the search schedule clearly defined and justified? (‘Living Systematic Review’ or a variation of this term should be included in the title.)

Not applicable

Are sufficient details of the methods and analysis provided to allow replication by others?

Yes

Are the conclusions drawn adequately supported by the results presented in the review?

Yes

Reviewer Expertise:

MIS, Artificial Intelligence, HCI, Cybersecurity, Business and IT alignment

I confirm that I have read this submission and believe that I have an appropriate level of expertise to confirm that it is of an acceptable scientific standard, however I have significant reservations, as outlined above.

# Response

**Published:** 2026-04-18

**Authors:** Hasan Naji

**Affiliations:** Computer Science, University of Mosul College of Computer Sciences and Mathematics, Mosul, Nineveh Governorate, Iraq

We sincerely thank the reviewer for the careful reading of our manuscript and for the constructive and insightful comments. We also apologize for the delay in submitting our revised response, which was due to a temporary health issue. We greatly appreciate your patience and understanding.

 In response to the reviewer’s comments, we have revised the manuscript in three main ways. First, we strengthened the synthesized discussion of the quantitative findings by expanding the interpretation of the reported FAR, FRR, EER, and accuracy values, with greater emphasis on the conditions under which particular biometric approaches perform well or show limitations. Second, we revised the usability and privacy discussion to more explicitly connect these challenges with the real-world implementation strategies used by major banks, particularly the use of biometrics together with OTP, device recognition, app-based approval, secure tokens, and step-up authentication. Third, we strengthened the conclusion and future directions by adding a clearer recommendation for more standardized evaluation protocols in future primary studies to support stronger comparative synthesis in subsequent reviews.

 We are grateful for the reviewer’s positive overall assessment of the manuscript and believe that these revisions have improved its clarity, depth, and practical relevance.
